# Tripalmitin nanoparticle formulations significantly enhance paclitaxel antitumor activity against breast and lung cancer cells *in vitro*

**DOI:** 10.1038/s41598-017-13816-z

**Published:** 2017-10-18

**Authors:** María Carmen Leiva, Raúl Ortiz, Rafael Contreras-Cáceres, Gloria Perazzoli, Iryna Mayevych, Juan Manuel López-Romero, Francisco Sarabia, Jose Manuel Baeyens, Consolación Melguizo, Jose Prados

**Affiliations:** 1 Institute of Biopathology and Regenerative Medicine (IBIMER), Center of Biomedical Research (CIBM), University of Granada, 18100 Granada, Spain; 2 Department of Anatomy and Embryology, Faculty of Medicine, University of Granada, 18071 Granada, Spain; 3 Biosanitary Institute of Granada (ibs. GRANADA), SAS-Universidad de Granada, 18014 Granada, Spain; 4 Department of Health Science, University of Jaén, 23071 Jaén, Spain; 5 Department of Organic Chemistry, Faculty of Science. University of Málaga, 29071 Málaga, Spain; 6 Department of Pharmacology, Institute of Neuroscience, Biomedical Research Center (CIBM), University of Granada, 18100 Granada, Spain

## Abstract

Paclitaxel (PTX) is one of the drugs of choice in the treatment of breast and lung cancer. However, its severe side effects, including mielosuppression, cardiotoxicity and neurotoxicity, frequently cause treatment to be discontinued. Solid lipid nanoparticles (NPs) of glyceril tripalmitate (tripalmitin) loaded with PTX (Tripalm-NPs-PTX) including modifications by the addition of hexa(ethylene glycol), β-cyclodextrin and macelignan were developed. All NPs-PTX formulations displayed excellent hemocompatibility and significantly enhanced PTX antitumor activity in human breast (MCF7, MDAMB231, SKBR3 and T47D) and lung (A549, NCI-H520 and NCI-H460) cancer cells. Tripalm-NPs-PTX decreased PTX IC_50_ by as much as 40.5-fold in breast and 38.8-fold in lung cancer cells and Tripalm-NPs-PTX macelignan inhibited P-glycoprotein in resistant tumor cells. In addition, Tripalm-NPs-PTX significantly decreased the volume of breast and lung multicellular tumor spheroids that mimics *in vivo* tumor mass. Finally, Tripalm-NPs-PTX decreased the PTX IC_50_ of cancer stem cells (CSCs) derived from both lung and breast cancer cells (6.7- and 14.9-fold for MCF7 and A549 CSCs, respectively). These results offer a new PTX nanoformulation based on the use of tripalmitin which improves the antitumor activity of PTX and that may serve as an alternative PTX delivery system in breast and lung cancer treatment.

## Introduction

Paclitaxel (PTX) has been proved to have excellent antitumor properties against breast and lung cancer. The action mechanism of PTX involves microtubule polymerization with mitotic cell arrest at the metaphase/anaphase that inhibits cell proliferation. Nevertheless, this beneficial antitumor activity is accompanied by the appearance of severe side effects such as mielosuppression, cardiotoxicity and neurotoxicity from peripheral neuropathy causing the treatment of the patient to be discontinued^1,2^. In addition, the presence of Cremophor EL in the PTX formulation, which is necessary because of the low drug solubility, causes serious adverse effects, such as hypersensitivity reactions. Finally, other PTX drawbacks include long infusion times, low drug concentrations in the tumor, poor bioavailability, and the development of multidrug resistance (MDR)^3^. To address these drawbacks, PTX was incorporated into nanoparticles (NPs) which were able to increase drug solubility avoiding the use of toxic solvents, protect against drug opsonization, metabolization and excretion and increase its bioavailability and accumulation in the tumor^3–5^. For example, albumin NPs associated with PTX (Abraxane), a nanoformulation with numerous advantages over current standard chemotherapy, have been approved by the U.S. Food and Drug Administration (FDA) for treating patients with various cancers, including metastatic breast cancer^4^. In this context, new PTX NPs formulations that could be used to improve the prognosis of cancer patients are still of great therapeutic interest.

Solid lipid NPs are of special interest to modify the pharmacokinetic and physicochemical properties of antitumor drugs due to their demonstrable biocompatibility, physical stability and scalability. In addition, these NPs may be functionalized to actively target cells and allow controlled drug delivery^6–8^. In particular, tripalmitin NPs (Tripalm-NPs) have already been used to deliver 5-fluorouracil^9^, tamoxifen citrate^10^ and curcumin^11^ with a high entrapment efficiency. Recently, the incorporation of sorafenib into Tripalm-NPs enhanced its antitumor activity in hepatocarcinoma cells^12^. Clearly, Tripalm-NPs can be conjugated with antibodies to improve the efficacy of the anticancer drug, as recently demonstrated^13^ using melanotransferrin antibody and etoposide against glioblastoma multiforme. Nevertheless, few articles have used PTX-loaded tripalmitin carriers. Previously, Cavalli *et al*.^14^ used tripalmitin and phosphatidylcholine (PC) NPs to deliver PTX and thus avoid its precipitation. Serpe *et al*.^15^ used PTX-loaded Tripalm-NPs to eliminate Cremophor EL in the formulation, although a similar antitumor effect to that of the free drug was observed. Further, PTX-loaded Tripalm-NPs have been used for the treatment of brain cancer since this drug is not able to cross the blood-brain barrier^16^. The recent development of new formulations of solid lipid NPs including tripalmitin are increasing the possibilities for transporting low solubility drugs as recently demonstrated Bondi *et al*.^12^ with the antitumor agent sorafenib.

Solid lipids NPs may be modified by the use of molecules such as hexa(ethylene glycol) (OEG), macelignan (MAC) or β-cyclodextrin (β-CD) which may provide new physicochemical characteristics with clinical applications. In fact, ethylene glycol modification of NPs may decrease their interaction with serum protein and prevent macrophage opsonization^17^. In addition, the incorporation of polyethylene glycol (PEG) onto solid lipid NPs has been used to stabilize tripalmitin NPs. In addition, the incorporation of polyethylene glycol (PEG) onto solid lipid NPs has been used to stabilize the system.^18^ Zhen *et al*.^19^ conjugated PTX-loaded solid lipid NPs with a PEGylated peptide that specifically interact with matrix metalloproteinase that is over-expressed by some tumour cells. Ethylene glycol derivatives also increased the cell internalization of monostearin solid lipid NPs with PTX in A549 lung tumours^20^. Solubility of hydrophobic drugs such as PTX has been improved using βCD. Lipophilic drugs form inclusion complexes when incorporated into the βCD lipophilic cavity, whereas the outer surface presents hydrophilic behavior^21^. In addition, the incorporation of PTX into βCD NPs displayed a high degree of physical stability and good hemocompatibility in comparison with common PTX solvents, and an increase in their antitumor activity against breast cancer cells^22^. Cyclodextrins have also been combined with several NPs forming ternary complexes to improve their biological properties. Recently, Baek and Cho^23^ modified solid lipid PTX-loaded NPs through the incorporation of 2-hydroxypropyl-βCD resulting in an increased drug internalization and cell death in the resistant MCF-7/ADR breast cancer cell line and a decrease in drug toxicity after intravenous injection. Finally, MAC, a natural ligand extracted from Myristica fragrans inhibits P-glycoprotein (P-gp), an efflux pump able to eject the drug once it has entered the tumor cells^24^. Interestingly, P-gp is one of the multidrug resistance (MDR) mechanisms responsible for PTX treatment failure^25^. It has been demonstrated that concomitant use of PTX and MAC improve the cellular accumulation of this antitumor drug by the inhibition of P-gp^26^. In addition, P-gp efflux pump may be blocked by different types of NPs, including solid lipid NPs^25,27^.

Our work represent the first study of a glyceryltripalmitate solid lipid NPs designed as a paclitaxel delivery system (Tripalm-NPs-PTX) to the breast and lung cancer treatment including assays in culture cells, multicellular tumor spheroids and cancer stem cells. The main objective of this study was to develop, characterize and assay PTX-loaded glyceryl tripalmitate solid lipid NPs (Tripalm-NPs-PTX) to significantly increase their antitumor effect and permeability in comparison to the free drug in both breast and lung human cancer cells cultures and multicellular tumor spheroids (MTS), an experimental system that mimics tumours *in vivo*. In addition, we modified Tripalm-NPs using OEG, a pure and non-dispersed oligomer as alternative to PEG (Tripalm-NPs-OEG), βCD (Tripalm-NPs-βCD) and MAC (Tripalm-NPs-MAC) to determine possible benefits in the PTX antitumor activity. Finally, we tested the effect of Tripalm-NPs-PTX against cancer stem cells (CSCs) derived from both lung and breast cancer cells. Our results showed that the new PTX-loaded solid lipid NPs improve the PTX effect in comparison to the free drug not only in culture cells and MTS but also in CSCs derived from both type of tumors.

## Results and Discussion

### Morphology of PTX-loaded tripalmitin NPs

**Figure 1:**
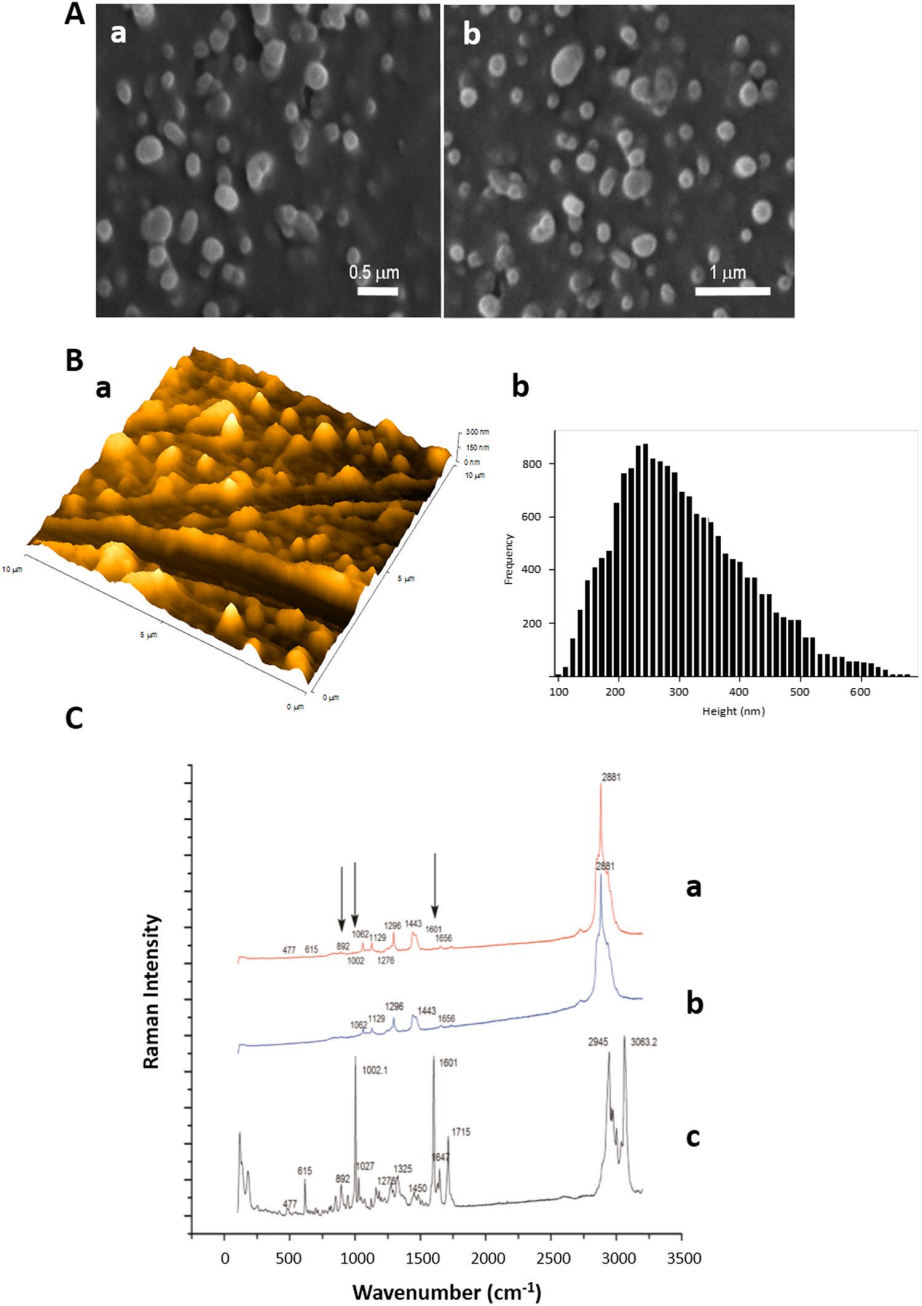
Tripalmitin NP-loading PTX morphology, size and chemical characterization. (**A**) Analysis by SEM microscopy revealing size in the range 210–220 nm: (a) micrograph with × 30.000 times magnification and (b) micrograph with × 20.000 magnification. (**B**) AFM microscopy: (a) topographic image at 10 µm scan range in 3D topographic configuration, and (b) height distribution histogram: analysis shows diameters of around 250 nm. (**C**) Raman spectra of a sample of Tripalmitin NP-loading PTX (a), of a sample of Tripalmitin NP (b) and of a pure sample of PTX (c).

PTX-loaded Tripalm-NPs can be prepared by following different methodologies including hot and cold homogenization and solvent microemulsion^28^. Preparation of solvent emulsions of tripalmitin/PTX by ultrastirring was chosen for this study since it allows a good control of the NPs size and a high degree on monodispersity^29^. In comparison to similar procedures, adding PTX to the mixture of melted tripalmitin and L-α-PC at 70 °C improves the active trapping by the lipid core of the NPs, increasing not only the percentage of incorporation of PTX to the NP, but also the stability of the hybrid material. Tripalm-NPs-PTX showed an average diameter of around 190 ± 7 nm by Z-Average Size analysis (Supplementary Fig. S1). Morphology analysis by SEM revealed a NPs size in the range of 210–220 nm (Fig. 1A). Further morphology analysis was carried out by AFM which indicated an average size of 250 nm as can be observed in the analysis of the height distribution histogram (Fig. 1B). The NPs size obtained by AFM was slightly larger in comparison to the Z-Average Size and SEM results. This can be attributed to the tip broadening which occurs when the cantilever tip is in contact with soft, sticky materials and to the surface effect^30^. SEM pictures show an anisometric spherical shape in the Tripalm-NPs-PTX with little aggregation of individualized particles. This effect has been attributed to the lipid nature of the carriers, surfactants and sample preparation prior to SEM analysis, while the spherical and non-spherical morphologies have been previously reported and attributed to the nature of the lipid carriers, their purity and to the lipid structure modification during the drying process prior to measurement^31,32^. Additionally, no roughness is observed on the particle surface. It is important to note that similar morphological results were found for the OEG, βCD, MAC and FITC modified Tripalm-NPs-PTX (see Methods) confirming that these additives do not affect NPs morphology. This fact can be attributed to the small percentage of additives in comparison to the main lipid component and surfactants. Therefore, as expected, lipid and surfactant concentrations determine the morphological characteristics. Moreover no important morphological differences have been found when the preparation of NPs was carried out either with 15 or 30 min (Supplementary Table S1) of homogenization.

Finally, Micro-Raman spectroscopy was used for the NP chemical composition analysis. Isolated NPs with a broad diameter were chosen because it allows deep chemical analyses. The presence of PTX loaded into NPs can be deduced by comparing pure PTX and tripalmitin bands with those of the Tripalm-NPs-PTX (Fig. 1C). The later Raman bands consist of the superposition of those for PTX and triplamitin. Because of the low concentration of PTX in the NPs only the most intense peaks of PTX can be detected. Line “a” corresponds to the Raman spectra of the NPs, which hardly differs from that of pure tripalmitin, whereas deeper analysis clearly shows the most intense PTX bands at 1002, 1601 and 1715 cm^−1^. These results confirm the incorporation of PTX into the particle. The PC contribution to the Raman spectrum is difficult to observe because the main band with a Raman contribution appears at 2950 cm^−1^ in this compound, which is the same frequency range as tripalmitin.

### Entrapment efficiency and drug loading

The HPLC technique shows values close to the total amount of PTX added to the NPs for their preparation, meaning a near total incorporation into the centrifuged Tripalm-NPs-PTX solid, and consequently the complete entrapment of PTX by the tripalmitin lipid matrix. These results of entrapment efficiency improve on those reported by different authors. In particular, Baek and Cho^28^ gave values as high as 79.0 ± 3.8% of encapsulation for PTX and docetaxel loaded into solid lipid NPs made of glyceryl behenate.

The quantitative incorporation of PTX into the NPs can be explained by the low drug loading value in the prepared Tripalm-NPs-PTX. Drug loading capacity is defined as the ratio between the weight of PTX in the NPs and the total NPs weight^33^. Drug loading in the prepared Tripalm-NPs-PTX is in the range of 0.4–0.03% (Supplementary Table S1), low enough to guarantee the complete incorporation of the PTX into the NPs. By way of example, common values have been reported of the PTX loading capacity of NPs of between 1.2 and 5.7% (w/w)^33^.

### ***In vitro*** release studies

**Figure 2:**
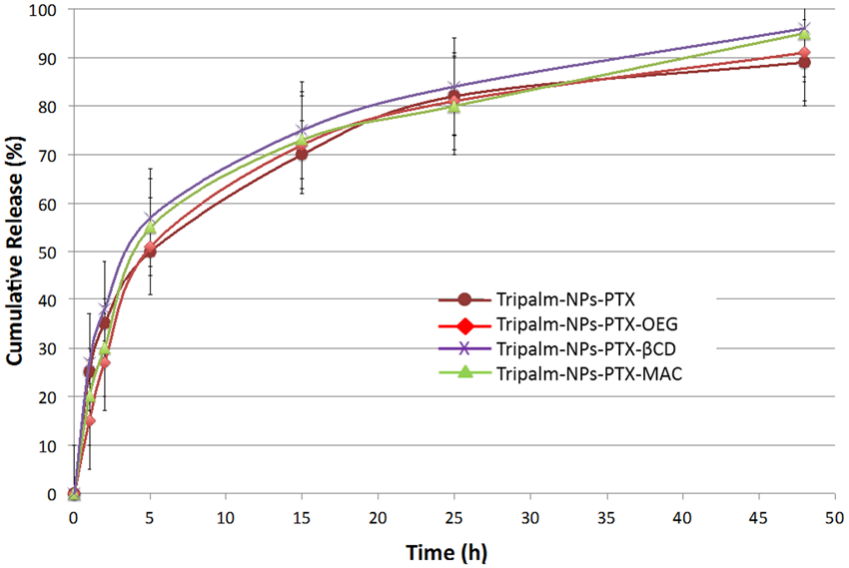
Tripalmitin NP-loading PTX entrapment efficiency and drug loading. Cumulative release of PTX from Tripalm-NPs at 37 °C by suspension of the samples in phosphate buffer solution and posterior extraction with ethanol. Quantities were measured by HPLC (see Methods). A slow release of PTX was found along the 48 h period, reaching in all samples 90% after 25 h. The data represents the mean value ± SD of quadruplicate experiences.

*In vitro* release was studied in pH controlled conditions (PBS, pH≈ 7.5) at 37 °C with slow shaking. Under these conditions, NPs degradation and liberation of PTX is promoted. PTX has been reported to have an aqueous solubility of 0.7–30 µg/mL. Therefore, to maintain sink conditions, PBS with 0.4% TW was used as the release medium. The solubility of PTX in the release medium at room temperature was 10.8 ± 0.3 µg/mL. The cumulative release of PTX from Tripalm-NPs-PTX was calculated based on the total PTX released (Fig. 2). As expected, a slow release of PTX was found throughout the 48 h period, reaching 90% after 25 h. The release is faster during the first 5 h, while the final release rates were much lower. The initial rapid release phase revealed that some of the drug was on or near the surface of NPs, and the second slow release phase might be caused by PTX release from the inner core of the NPs. Similar results were found when a liberation study was carried out with the additive modified NPs samples Tripalm-NPs-PTX-OEG, Tripalm-NPs-PTX-βCD and Tripalm-NPs-PTX-MAC (Fig. 2), meaning that the different cytotoxic activities found, can not be attributed to different PTX release patterns.

### Tripalmitin NPs hemocompatibility

Tripalmitin-based NPs including modified NPs (Tripalm-NPs-MAC, -OEG and -βCD) showed little disruption of human erythrocytes when the hemolysis assay was carried out (Supplementary Fig. S2A). In fact, in comparison to Triton X-100 exposure, which was used as a control (100% hemolysis), none of the NPs induced more than 10% hemolysis, even at the highest dose tested. Tripalm-NPs and Tripalm-NPs-MAC were shown to be the least toxic delivery systems. In addition, erythrocyte morphology after exposure to Tripalm-NPs and modified NPs at the highest concentration was tested, showing no significant alteration (Supplementary Fig. S2B).These results demonstrate the hemocompatibility of all tripalmitin. NPs assayed and support the previously demonstrated suitability of the solid lipid NPs for *in vivo* administration^7,34^.

### PTX-loaded tripalmitin NPs improve drug cytotoxicty in breast and lung cancer cells

**Figure 3:**
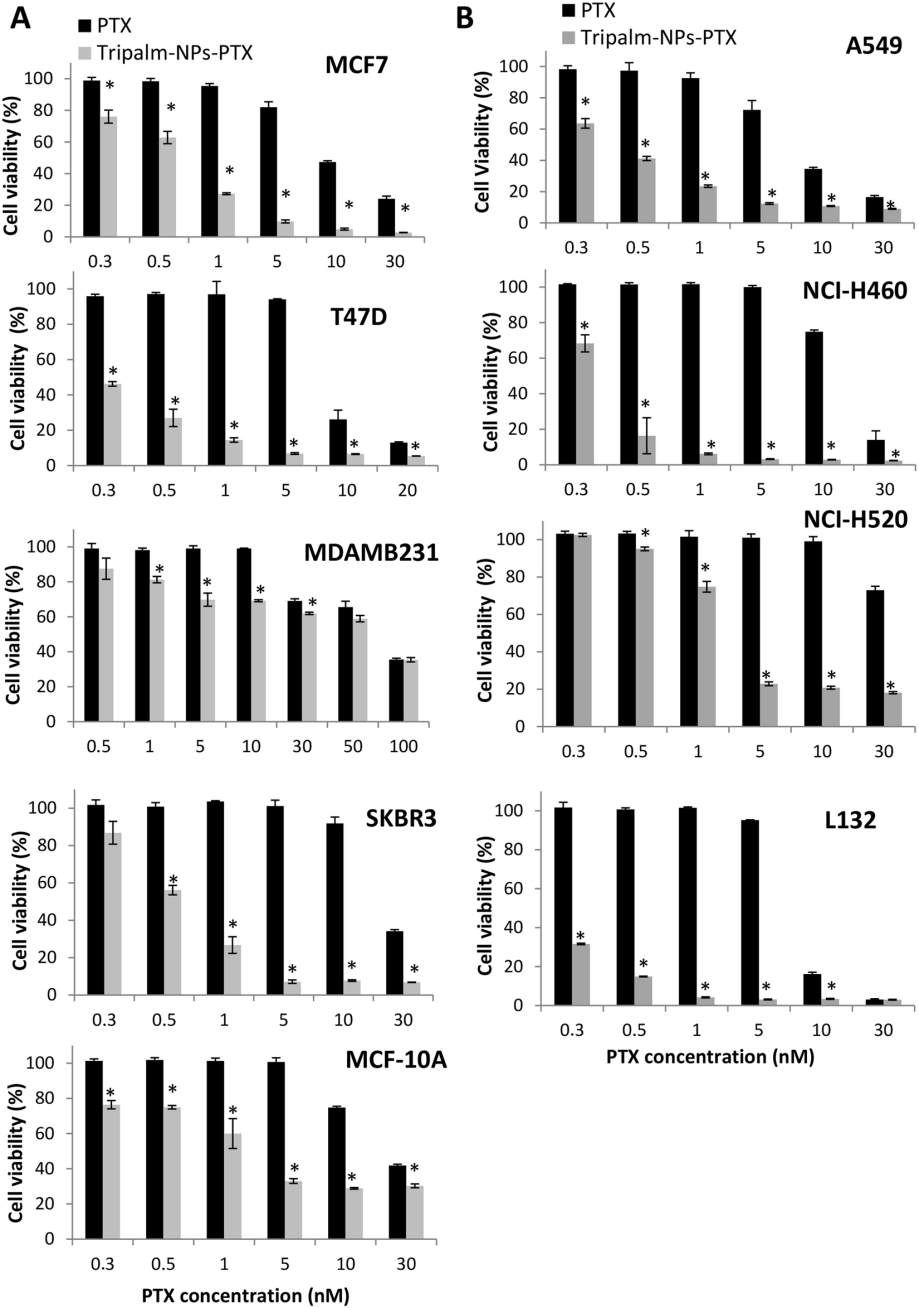
Cytotoxicity of Tripalm-NPs-PTX. Cell viability (%) of human breast tumor and normal cells (**A**) and human lung tumor and normal cells (**B**) was tested after treatment with Tripalm-NPs-PTX and free PTX. Cells were treated with Tripalm-NPs-PTX or free PTX for 96 h. Data represent the mean value ± SD of quadruplicate cultures. (*) Significant differences (p ≤ 0.001) between free PTX and Tripalm-NPs-PTX.

Tripalm-NPs with no drug induced no variation in the percentage of cell viability in all breast and lung cancer cell lines (Supplementary Fig. S3). By contrast, PTX carried by Tripalm-NPs significantly increased the percentage of relative inhibition after 96 h of exposure in both breast and lung cancer cell lines in relation to free PTX (p < 0.001). In particular, a significant PTX IC_50_ decrease was observed in T47D, SKBR3 and MCF7 breast cancer cell lines (40.5-, 39.8-, and 18.5-fold, respectively) in relation to free drug (Fig. 3A). Only the MDAMB231 breast cancer cells showed a similar IC_50_ value when Tripalm-NPs-PTX and PTX were used. Interestingly, MDAMB231 cells displayed significantly enhanced cell death with low Tripalm-NPs-PTX doses (p < 0.001) which may be related to the cell line subtype (triple negative for ER, PR and HER-2) and its biological characteristic resembling tumor stem cells which are associated with a poor cancer prognosis^35^. PTX IC_50_ also decreased in the A549, NCI-H520 and NCI-H460 lung cancer cell lines (19.8-, 18.7- and 38.8- fold, respectively) when where treated with Tripalm-NPs-PTX (Fig. 3B). Tripalm-NPs-PTX showed also toxicity against inmortalized epithelial cells used as a control. However, non tumor breast and lung cells behaved differently. Whereas the non tumoral breast MCF-10A cells showed only an 11.2-fold PTX decrease in IC_50_ after Tripalm-NPs-PTX exposure, non tumoral L132 lung cells showed a PTX IC_50_ decrease of nearly 39-fold. So, Tripalm-NPs-PTX was more toxic in cancer cells than in MCF10A normal cells (with the exception of MDAMB231 cell line). By contrast, NPs-PTX showed a similar toxicity in the L132 normal cells than in NCI-H460 and A549 lung cancer cells (Fig. 3A and B). This control cells toxicity has been observed in similar studies using the same inmortalized cells^36,37^. In addition, our results contrast with those found in HT-29 colorectal cancer cells in which PTX-loaded tripalmitin NPs achieved rates of cell death similar to free PTX^15^ although the avoidance of Cremophor in this formulation provided an advantage in itself. Yuan *et al*.^20^ compared the cytotoxic effect of different PTX-loaded solid lipid NPs on A549 lung tumor cells, monostearin NPs being the most promising ones. In this case, cell death was related to the NPs uptake pattern. Trimyristin NPs have also been tested on MCF7 breast and OVCAR-3 ovarian human cancer cell lines, and cytotoxicity was similar to the commercial PTX formulation based on Cremophor EL on both cell lines^18^. Recently, PTX-loaded mannosylated-distearoyl-phosphatidyl-ethanolamine solid lipid NPs caused greater cell death than free PTX (54.4% vs 42.8% at 40 mg/mL) after 48 h of treatment without causing any toxicity in A549 lung cancer cells. In this case, the use of mannose as a lecitin receptor ligand increased the anticancer activity of PTX against A549^38^. These NPs have recently been modified by using mannose for active targeting against lung A549 cancer cells.

### Modified PTX-loaded tripalmitin NPs drug cytotoxicity in breast and lung cancer cells

In order to analyze the antitumor effect of modified Tripalm-NPs (Tripalm-NPs-MAC-PTX, -OEG-PTX and -βCD-PTX) against breast and lung cancer cells we selected both MCF7 and A549 cells to carry out cytotoxic tests. Normal MCF-10A and L132 cells were used as a control. As shown in Fig. S4, neither of the modified NPs achieved a significantly higher antitumor effect than non-modified Tripalm-NPs-PTX. Only Tripalm-NPs-OEG-PTX induced a PTX IC_50_ decrease (15.9-fold), this being similar to that found with Tripalm-NPs-PTX in MCF7. Blank modified Tripalm-NPs when assayed in both normal and tumoral cells displayed no cytotoxicity (data no shown) like Tripalm-NPs. However, all modified Tripalm-NPs significantly improved the antitumor effect of PTX in relation to the free drug in both breast and lung cancer (p < 0.001) (Supplementary Fig. S4). So, despite the fact that the use of OEG, MAC and βCD does not directly increase the Tripalm-NPs-PTX *in vitro* cytotoxicity, they could provide new biological characteristics that improve their antitumor activity *in vitro* or *in vivo*. In fact, PEG and OEG, widely used to modify solid lipid NPs, seem to increase antitumor specificity and activity. Recently, Zheng *et al*.^19^ demonstrated a significant reduction in tumor growth and increase in survival times in C57BL/6 N mice with induced tumors treated with PEGylated NPs. Our results showed that the MCF7 therapeutic index after Tripalm-NPs-OEG-PTX treatment was nearly 15.9-fold whereas in MCF-10A normal breast cells it was only 3.8-fold, suggesting that OEG may increase tumor specificity, producing a lower cytotoxic effect in non tumor cells. In addition, PEGylated NPs increased drug blood circulation times, and then, its bioavailability and treatment efficacy^39^. *In vivo* assays would be needed to confirm the possible biological advantage of these modified systems. In accordance with our results, Baek and Cho^23^ also showed that solid lipid NPs modified with hydroxypropyl-β-CD induced a similar cytotoxicity than non modified NPs although in their results an improvement of the PTX effect in solution was detected. Finally, Tripalm-NPs-MAC-PTX significantly increased the antiproliferative activity of the free PTX but not the Tripalm-NPs-PTX efficacy. Previous studies proved an improvement of PTX uptake in P-pg resistant cell lines with a combined administration of PTX and MAC^26^. More studies have been performed (see Modulation of drug resistance by PTX-loaded tripalmitin NPs) with a resistant cell line to explore this property.

### Cell internalization assay with PTX-loaded tripalmitin NPs

**Figure 4:**
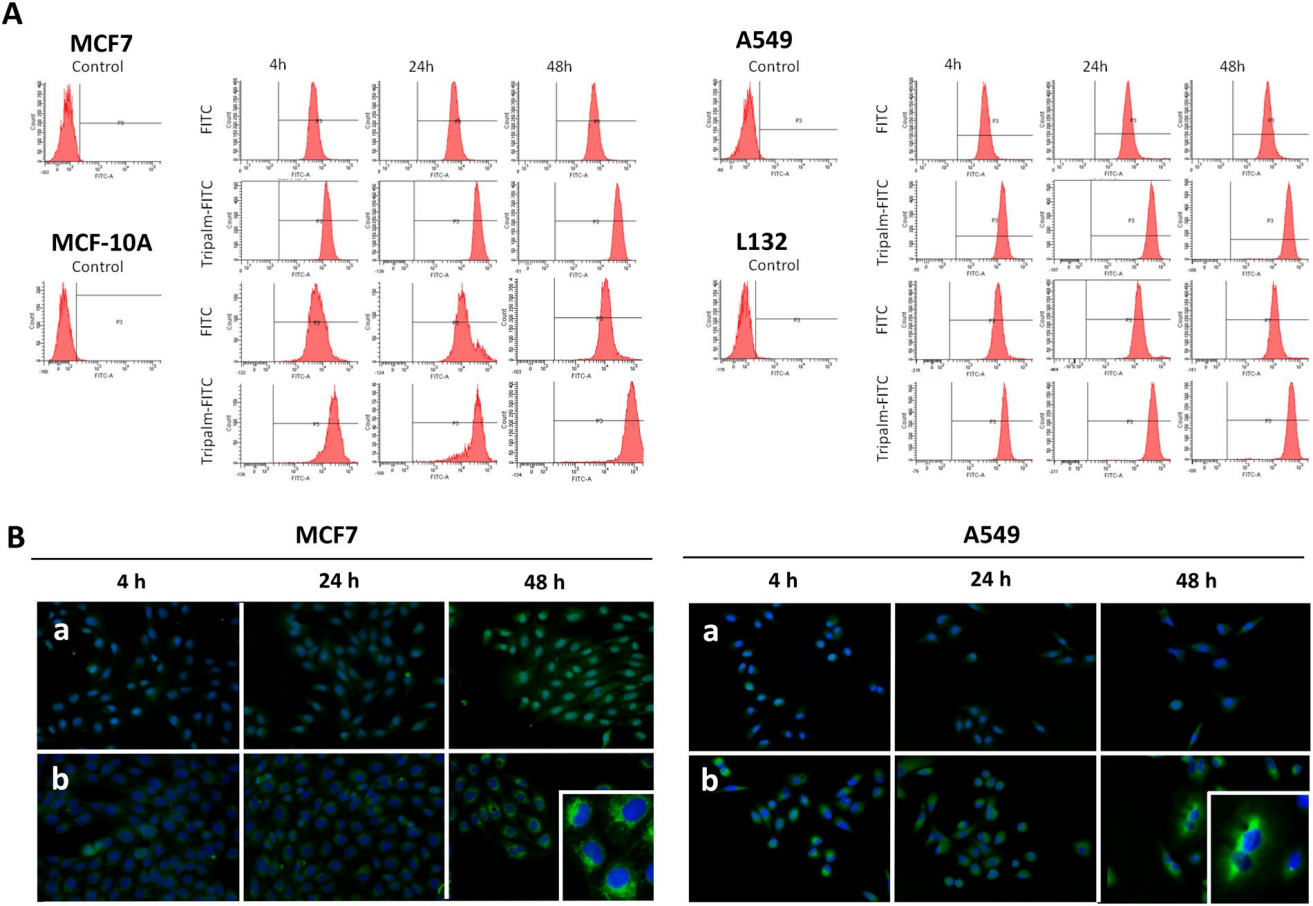
Analysis of Tripalm-NPs cell internalization. (**A**) FACScan analysis of the MCF7, MCF-10A, A549 and L132 cells exposed to Tripalm-NPs-FITC and FITC in solution at different exposure times. Untreated cells were used as control. (**B**) Representative fluorescent images from the FITC (a) and Tripalm-NPs-FITC (b) internalization in MCF7 breast and A549 lung tumor cell lines at different times. Nuclei were stained in blue, whereas FITC appeared in green. FICT in solution is accumulated in the cell nucleus since Tripalm-NPs-FITC were retained in the cytoplasm. Magnification: 40X.

Tripalm-NPs-FITC was used in order to assess their cell uptake through a flow cytometry assay. As shown in Fig. 4A, breast and lung tumoral and normal cells exhibited a similar time-dependent Tripalm-NPs-FITC internalization whereas FITC alone was barely internalized. Cell uptake results were corroborated in MCF7 and A549 by an inmunofluorescence assay (Fig. 4B). Interestingly, a different fluorescence pattern was observed after Tripalm-NPs-FITC and FITC exposure showing predominance in the cell cytoplasm and nuclei, respectively. These results suggest that Tripalm-NPs do not produce nuclear permeability.

In addition, HPLC analysis demonstrated a significant increase of PTX concentration into cells when the Tripalm-NPs were used (Supplementary Fig. S5). Fan *et al*.^40^ showed that tripalmitin NPs improved cell uptake in Caco-2/HT29-MTX co-cultured cells suggesting a clathrin-dependent or caveolae-dependent endocytosis. Rivolta *et al*.^41^ proposed a similar mechanism mediated by the plasma membrane exchange as well as by active endocytosis, to a minor extent. However, at the moment the tripalmitin NPs internalization mechanism is not clear.

### Modulation of drug resistance by PTX-loaded tripalmitin NPs

**Figure 5:**
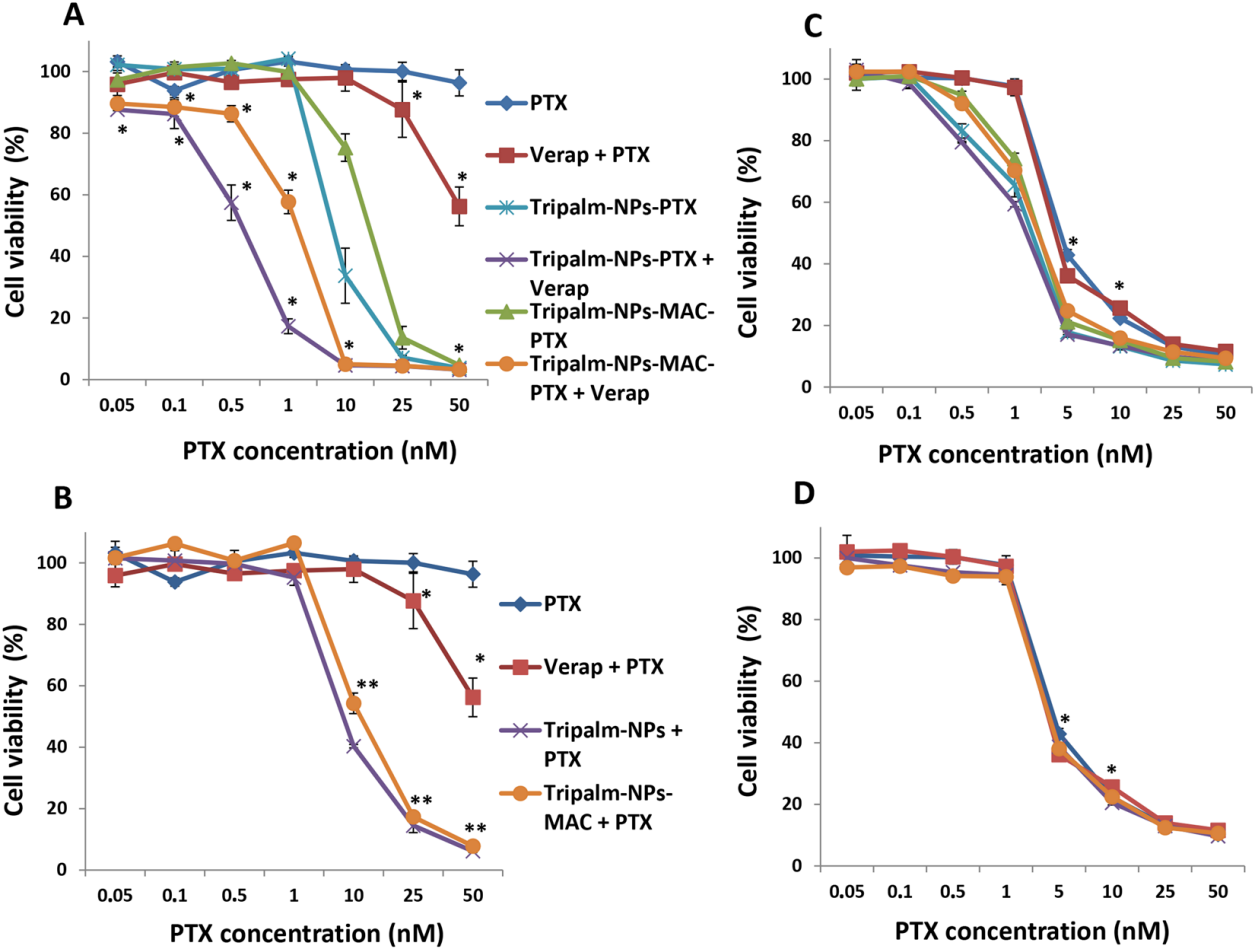
Cytotoxicity of Tripalm-NPs-PTX and Tripalm-NPs-MAC-PTX in resistant HCT-15 and sensitive T84 cell lines. HCT-15 cells were exposed to Tripalm-NPs-PTX and Tripalm-NPs-MAC-PTX and their combination with Verapamil (P-gp inhibitor) (**A**) and Tripalm-NPs and Tripalm-NPs-MAC (blank NPs) and their combination with PTX in solution (**B**). T84 cells, used as a control, were exposed to the same treatments (**C** and **D**, respectively). In both experiences PTX and PTX + Verapamil were assayed. Verapamil and blank NPs were added in a concentration equivalent to the 50 nM dose 24 hours before. Cells were treated with combination of drugs for 96 h Data represent the mean value ± SD of quadruplicate cultures. (*) Significant differences (p ≤ 0.001) between treatments with and without Verapamil. (**) Significant differences (p ≤ 0.001) between PTX and PTX + blank NPs treatments.

As mentioned above, P-gp may be implicated in the resistance to PTX. Following Baek and Cho^28^ and in order to demonstrate the ability of Tripalm-NPs-PTX to inhibit P-gp, a cytotoxic assay using a P-gp resistant HCT-15 cell line was carried out. The T84 cell line which did not overexpress P-gp was used as a control. In addition, the modified Tripalm-NPs with MAC, a P-gp inhibitor, was assayed. None of the NPs tested showed toxicity in these cell lines (data no shown). Both HCT-15 and T84 cells were treated with free PTX, Tripalm-NPs-PTX, Tripalm-NPs-MAC-PTX and their combination with the P-gp inhibitor Verapamil (Fig. 5).

Free PTX barely had any effect on HCT-15 (less than 10% of cell viability at the highest dose) although its association to verapamil improved the PTX IC_50_ (52.0 ± 3.1 nM). Interestingly, Tripalm-NPs-PTX significantly (p < 0.001) increased the PTX antitumor effect on these resistant cells, reaching an IC_50_ of 7.4 ± 1.0 nM. Furthermore, the combined treatment Tripalm-NPs-PTX + Verapamil increased the cytotoxic effect even further by reducing IC_50_ up to 0.5 ± 0.1 nM (14.8-fold decrease) (Fig. 5A). In addition, Tripalm-NPs-MAC-PTX also reduced PTX IC_50_ both when unassociated and associated with Verapamil (14.2 ± 0.6 nM and 1.4 ± 0.3 nM, respectively) but did not improve cytotoxicity in HCT-15 resistant cells with respect to non modified NPs. According to these results, MAC did not improve cytotoxicity in the resistant cell line (Fig. 5A). In addition, as shown Fig. 5B, the administration of free PTX along with blank NPs achieved a similar effect to PTX-loaded NPs supporting the capacity of the NPs themselves to modulate resistance. Only a slight decrease in the IC_50_ value was observed with the use of Tripalm-NPs-MAC + PTX (11.1 ± 0.9 nM) with respect to its PTX-loaded NPs-MAC (14.2 ± 0.6 nM) indicating that the NPs surface modification with MAC did not have any impact on the blockage of the P-gp resistance mechanism (Fig. 5B). On the other hand, the free PTX IC_50_ of the T84 non drug resistant cell line (4.6 ± 1.0 nM) was also significantly improved by both Tripalm-NPs-PTX (IC_50_ = 1.7 ± 0.2 nM) and Tripalm-NPs-MAC-PTX (IC_50_ = 2.3 ± 0.3 nM), resulting in a reduction of 2.7- and 2-fold respectively (Fig. 5C). However, in contrast to HCT-15, the association with Verapamil did not improve the effect of any of the previous treatments, probably owing to the low P-gp expression in this cell line. In fact, this low P-gp expression may explain why the combined administration of PTX and blank NPs did not show any differences in relation to the free PTX effect (Fig. 5D). According to these results, inhibition of P-gp plays an important role in the efficacy of these NPs against resistant cell lines.

**Figure 6:**
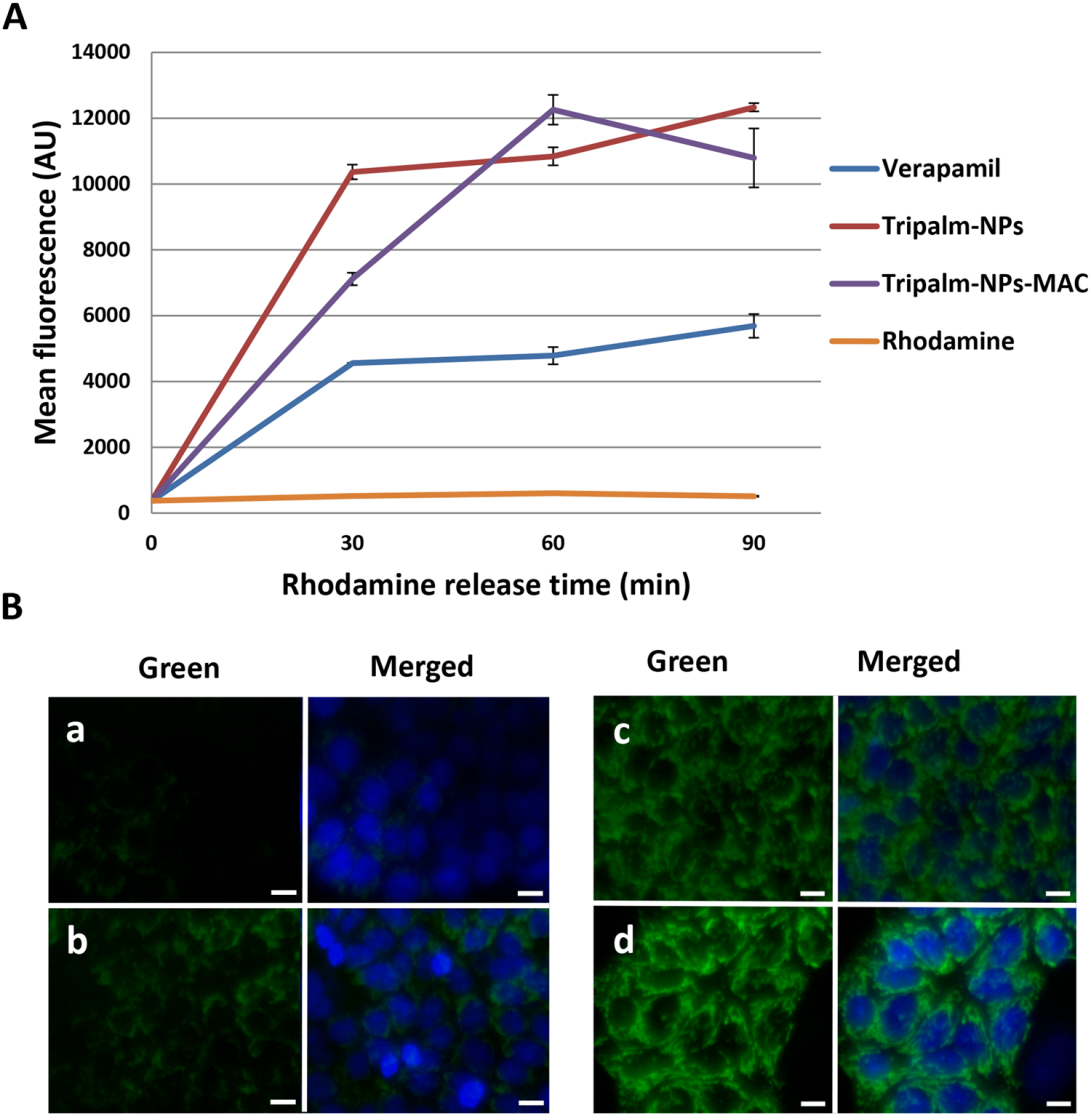
Rhodamine assay in resistant HCT-15 cell line. (**A**) Flow cytometry analysis after exposure of HCT-15 cells to Rhodamine and Rhodamine together with Verapamil, Tripalm-NPs and Tripalm-NPs-MAC. Verapamil and NPs were added 24 hours before the addition of Rhodamine. Rhodamine retention was measured at different times. Data represent the mean value ± SD of quadruplicate experiences. Mean fluorescence are expressed in arbitrary units (AU). (**B**) Representative fluorescent images of Rhodamine retention into HCT-15 cells nonpretreated (a) and pretreated with Verapamil (b), Tripalm-NPs (c) and Tripalm-NPs-MAC (d). Pretreatment was carried out 24 hours before Rhodamine exposure. Rhodamine was observed in green. Nuclei were stained in blue. Scale bar = 10 µm.

To confirm P-gp inhibition by NPs, a Rhodamine retention assay by flow cytometry and microscopy was carried out. As shown in Fig. 6, Rhodamine was immediately ejected from the HCT-15 cells, whereas Verapamil promoted Rhodamine retention. Both Tripalm-NPs and Tripalm-NPs-MAC increased Rhodamine accumulation in the cells to a similar extent, but much more effectively than Verapamil (up to 2-fold), as demonstrated fluorescent microscopy analysis (Fig. 6). Hence, our findings suggest that both NPs highly inhibit P-gp to a similar extent (and more than Verapamil), preventing drug expulsion and increasing PTX activity in tumor cells.

Several NPs have already shown an enhanced Rhodamine accumulation in P-gp overexpressing cells^42^. Recently, solid lipid NPs loading PTX showed a significant improvement of PTX cytotoxicity in the MCF-7/ADR breast cancer resistant cell line^28^. In fact, whereas cell viability after free PTX treatment (1 µM) was 65%, NPs cell exposure decreased this value by as much as 36.5%. Acetyl alcohol/polysorbate-based NPs achieved a reduction of PTX IC_50_ (from 1000 nM to 360 nM) in HCT-15 cells and a decrease in tumor volume after treatment of HCT-15 xenografted mice^43^. On the other hand, MAC has been proved to enhance PTX uptake in P-pg resistant cells with promising results^26^, although in our delivery systems the P-gp inhibitory effect may be related to the Tripalm-NPs formulation itself.

### Tripalm-NPs-PTX increase apoptosis in MTS from breast and lung cancer cells

**Figure 7:**
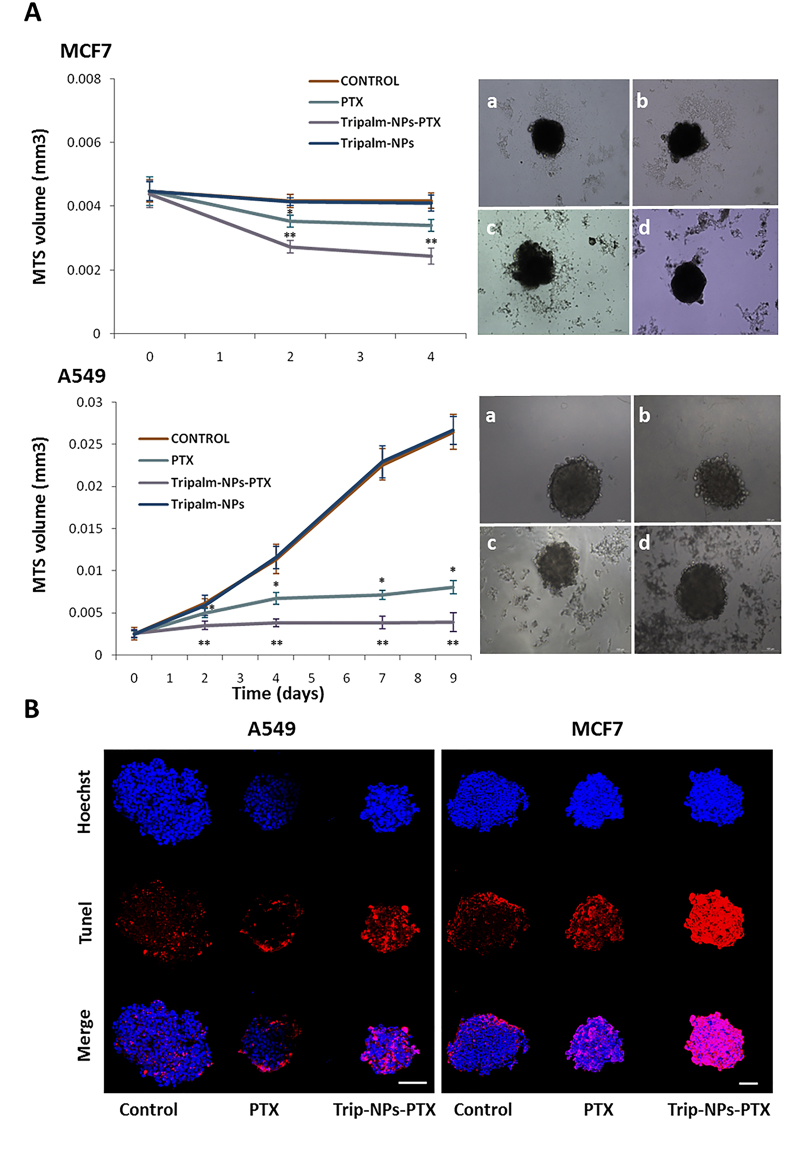
Analysis of MTS after Tripalm-NPs-PTX exposure. (**A**) Graphic representation of MCF7 and A549 MTS volumes (mm^3^) monitorization at different times of the experiment. MTS volume was calculated by the measurement of the shortest and longest diameter (see Methods). Data represent the mean value ± SD of 8 replicates. (*) Significant differences (p ≤ 0.001) comparing PTX and controls. (**) Significant differences (p ≤ 0.001) comparing PTX and Tripalm-NPs-PTX. At the right, representative images (at day 4) of MTS from MCF7 and A549 cells without treatment (controls) (a) and treated with PTX (b), Tripalm-NPs-PTX (c) and Tripalm-NPs (d). (**B**) Representative images of the apoptosis induced by Tripalm-NPs-PTX and free PTX on MTS from MCF7 and A549 cells at day 4 of the treatment. Apoptosis (in red) was detected using a TUNEL assay. MTS were treated with free PTX and Tripalm-NPs-PTX at a dose equivalent to the IC_50_ value of PTX (see Methods). Non treated MTS were used as controls. Nuclei were stained with Hoechst (blue). Original magnification: 40x; bar = 100 μm.

Tripalm-NPs-PTX were assayed in A549 and MCF7 MTS that is considered an ideal method for anticancer drug screening since they resemble tumor mass morphology^44^. Previously, it has been demonstrated that the modulation of A549 MTS after PTX treatment displays a dose-dependent behavior^45^. Monitoring A549 and MCF7 MTS showed a volume reduction significantly higher with Tripalm-NPs-PTX than with free PTX (p < 0.001) (Fig. 7A). Volumes in non-treated MTS were similar to those treated with blank Tripalm-NPs, demonstrating the lack of toxicity of these NPs. In addition, a TUNEL assay showed a loss of cell organization and a larger apoptotic area in both A549 and MCF7 MTS treated with Tripalm-NPs-PTX (Fig. 7B) suggesting an improve of penetration and/or antitumor activity of the drug that could represent a therapeutic advantage to the breast and lung cancer treatment.

### Cytotoxicity of Tripalm-NPs-PTX in cancer stem cells

**Figure 8:**
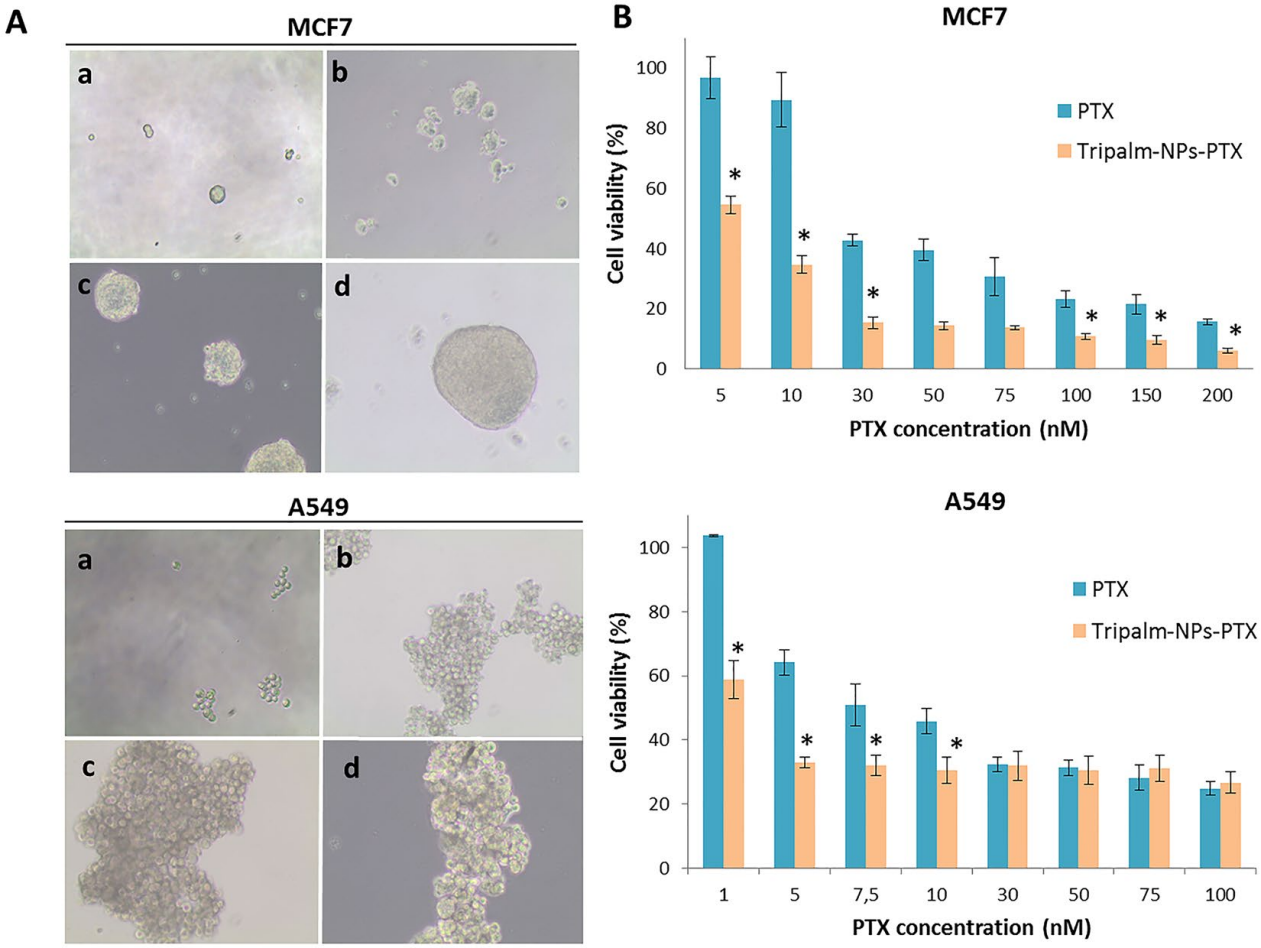
Cytotoxicity of Tripalm-NPs-PTX on CSCs obtained from MCF7 and A549 cells. (**A**) CSCs tumor spheres were observed since day 3 of incubation in a CSCs induction medium (see Methods). Photographs were taken at 1 (a), 3 (b), 6 (c) and 13 (**d**) days (magnification = 10X). (**B**) Cytotoxicity of PTX and Tripalm-NPs-PTX over CSCs from MCF7 and A549 was analyzed at different drug concentrations. Data represent the mean value ± SD of five replicates. (*) Significant differences (p ≤ 0.001) were observed when comparing Tripalm-NPs-PTX and PTX.

In order to demonstrate the activity of Tripalm-NPs-PTX against CSCs from breast and lung cancer, MCF7 and A549 cell lines were incubated in an induction medium (see Methods). Tumorsphere formation from both cell lines could be observed from the first days of testing (Fig. 8). The presence of specific CSCs markers in tumorspheres was assessed by quantitative real-time PCR analysis (Supplementary Fig. S6). After 72 h of treatment, Tripalm-NPs-PTX induced a higher cell death rate in both lung and breast CSCs in relation to free PTX, with a significant decrease in the IC_50_ (6.7- and 14.9-fold for MCF7 and A549 CSCs, respectively). This initial activity of the Tripalm-NPs-PTX over CSCs may be improved through the use of target molecules. In fact, the use of CD44 and CD133 in NP surfaces increased the therapeutic efficacy of the drug in these type of cells^46,47^. Further studies will be necessary to shed light on this subject.

## Conclusion

In conclusion, quantitative encapsulation of PTX in Tripalm-NPs was successfully achieved by the ultra-stirring method. Tripalm-NPs-PTX were around 210 nm in diameter and with little aggregation. Moreover, morphology and PTX release patterns of these NPs have been found not to be dependent on the additive content (OEG, βCD or MAC). What is more, all lipophilic Tripalm-NPs-PTX allow controlled and sustained drug release. Tripalm-NPs-PTX were able to significantly reduce the PTX IC_50_ on breast (up to 40.5-fold) and lung (up to 38.8-fold) cancer cells, providing a greater cytotoxic effect. This increased antitumor activity was corroborated using MTS, where Tripalm-NPs-PTX significantly decreased the volume of both breast and lung MTS. Modifications of the Triplam-NPs-PTX using OEG, βCD and MAC also improved the efficacy of PTX in both types of cancer cells but not in relation to the original Tripalm-NPs-PTX. Interestingly, Tripalm-NPs-PTX and NPs modified with MAC were able to significantly inhibit the efflux pump activity of the P-gp, although MAC did not induce a significant increase in P-gp blockage. Both modified and non-modified tripalmitin NPs showed high hemocompatibility. Finally, Tripalm-NPs-PTX induced a significant decrease in the PTX IC_50_ of cancer stem cells (CSCs) derived from both lung and breast cancer cell lines (6.7- and 14.9-fold for MCF7 and A549 CSCs, respectively). Our results demonstrate that the new PTX-loaded solid lipid NPs, based on the use of glyceryl tripalmitate and its modifications, significantly enhance antitumor activity in breast and lung cancer cells, as well as MTS and CSCs when compared to the free drug. These new nanoplatforms should be exploited as a new drug delivery system for improving current breast and lung cancer treatments.

## Methods

### Preparation, morphology analysis and chemical characterization of PTX-loaded tripalmitin NPs

Tripalm-NPs-PTX were prepared by following the ultrahigh-speed homogenization procedure. PTX (range 0.03–0.07%) was the active organic component and MAC or or βCD (0.03–0.2%) or OEG (0.1–0.6%) was added to some samples as the modifying agent (Tripalm-NPs-MAC-PTX, Tripalm-NPs-OEG-PTX and Tripalm-NPs-βCD-PTX). In addition, fluorescein isocyanate (FITC) (25 mg) was selected to develop Tripalm-NPs-FITC which was used in the internalization assay (supplementary Table S1). In a typical experiment, a 70 °C-preheated mixture of Tween® 80 (TW80) (0.85 g, 5.7%) and n-butane (nB, 0.17 g, 1.1%) was added to a magnetically stirred mixture of melted (70 °C) tripalmitin (0.3 g, 2.0%) and L-α-PC (0.15 g, 1.0%) followed by water (8 mL). Magnetic stirring was continued (5 min) and PTX (0.004 g) and the modifying agent (OEG, 20 mg) were added to the mixture. After 5 min more of magnetic stirring (70 °C), the mixture was dispersed with water (7.0 mL, 20 °C), and the o/w nanoemulsion obtained was ultrahigh-speed homogenized (SilentCrusher-M Homogenizer, Heidolph, Berlin, Germany) for between 15 to 30 min. After the homogenization period, the milky suspension was transferred to a glass vial, protected from light, and kept at 5 °C. Representative samples were taken to determine PTX-loaded NPs morphology by SEM and AFM, and diameter by Z-Average Size (Malvern Zetasizer Nano-ZS, supplementary section 1). Chemical characterization was carried out by micro-Raman spectroscopy (supplementary section 2).

### HPLC analysis: loading capacity and drug release of PTX-loaded tripalmitin NPs

HPLC analyses were carried out for the validation of the loading capacity of the NPs and the *in vitro* active release. A Thermo Spectrasystem apparatus equipped with a photodiode array detector was used. Separation was achieved on a Phenomenex C18 column (250 mm × 4.6 mm; 5 µm, Varian) operating at 25 °C. The mobile phase consisted of water: acetonitrile (60:40) for 40 min at a flow rate of 0.7 mL/min and an injection volume of 10 µL. PTX was detected at 227 nm. Previous to analysis, samples were filtered off through Teflon filter nozzles of 0.2 µm per syringe. Under these conditions, PTX was observed as separated and with a well-defined peak at around 5 min retention time that allowed the quantification. The calibration curve was prepared according to Sadeghi-aliabadi *et al*.^48^ To determine the amount of drug incorporated into the PTX-loaded Tripalm-NPs (loading capacity), 2 mL of the NPs emulsion was centrifuged (3000 rpm, 5 min). The aqueous layer (approx. 1.5 mL) was decanted and the remaining solid was extracted with ethanol (7 mL at 80 °C) for 5 min. The ethanolic extract was centrifuged (3000 rpm, 5 min) to eliminate solids, and the supernatant filtered off and injected into the HPLC system for quantitatively determining the amount of PTX in the ethanol phase, which in turn allows an estimation of the PTX loading in the NPs^49,50^.

To determine the PTX release from Tripalm-NPs-PTX, representative samples of NPs (2 mL) were centrifuged (3000 rpm, 5 min) in two test tubes provided with a cap. The aqueous layers were decanted and water (3 mL) was added to the solid, followed by centrifugation (3000 rpm, 5 min). This procedure was repeated one more time. After the final decantation, two samples of solid NPs were obtained (approx. 0.150 mg each). Both samples were suspended in phosphate buffer solution (PBS, 5 mL each, pH≈ 7.5) and TW80 (0.02 mL) and the tubes were placed in a water bath at 37 °C and shaken at 100 rpm. At specific intervals, the release medium containing the drug was transferred out and extracted with ethanol (2 × 3 mL). Fresh release media (5 mL) was added to the test tubes to continue the release studies. The ethanol extracts were allowed to evaporate and the residue was reconstituted in water/acetonitrile (3 mL, 60:40) for HPLC analysis.

### Cell culture and cytotoxicity study

To assess the antitumor efficacy of the PTX-loaded Tripalm-NPs we used human breast (MCF7, MDAMB231, SKBR3 and T47D) and lung (A549, NCI-H520 and NCI-H460) cancer cell lines. Normal breast (MCF-10A) and lung (L132) cell lines were used as a control. In addition, HCT-15 and T-84 colon carcinoma cell lines with high (resistant) and low (sensitive) P-gp expression, respectively, were used for the MDR inhibition study. All cell lines were purchased and grown following American Type Culture Collection (ATCC, Manassas, VA) recommendations. Cells were seeded in 24-well plates at different densities depending on the cell line, in 400 μl of culture medium and incubated overnight. Then, cells were treated with PTX-loaded Tripalm-NPs and free PTX at increasing concentrations to determine 50% inhibitory concentration (IC_50_) value (96 h). In addition, cells were exposed at the equivalent concentration of blank Tripalm-NPs.

Afterwards, a sulforhodamine B (SRB) colorimetric assay was performed as we previously described^51^. Optical density (OD) was measured with a Titertek multiscan colorimeter (Flow, Irvine, California) at 492 nm. The percentage of cell viability (cell viability %) was calculated as follows:

1 $$Cell\,viability\,( \% )=\frac{sample\,OD}{negative\,control\,OD}\times 100$$

2 $$Therapeutic\,index\,(fold)=\frac{PTX\,I{C}_{50}}{NPs-PTX\,I{C}_{50}}$$

The IC_50_ value was calculated with GraphPad Prism 6 using cell viability data.

### Hemocompatibilty assay

To demonstrate hemocompatibility of tripalmitin NPs, we used a modification of the hemolysis assay described by Evans *et al*.^52^ (supplementary section 3). The percentage of hemolysis was calculated with the following formula:

3 $$Hemolysis\,( \% )=\frac{abs.\,of\,the\,sample-abs.\,of\,the\,negative\,control}{abs.\,of\,the\,positive\,control}\,\times 100$$

### Cell internalization assay

Cell uptake was analyzed by flow cytometry in tumor (A549 and MCF7) and non tumor cells (L132 and MCF-10A). Cells were seeded in a 6-well plate at 45 × 10^3^ cells/well for A549, L132 and MCF-10A and 25 × 10^3^ cells/well for MCF7. Then, Tripalm-NPs-FITC (see previously) were added (21.6 µM) for 4, 24 and 48 h. Cells were centrifuged and resuspended in 200 µl of PBS. Fluorescence intensity of FITC (λ emission = 520 nm) was measured with a flow cytometer BD FACSCanto II (Becton Dickinson, San Jose, USA). In addition, MCF7 and A549 cell lines were seeded in 8-chamber Falcon™ Culture Slides (4 × 10^3^ and 6 × 10^3^ cells per well, respectively) and incubated overnight in complete DMEM. Treatments were the same as before. Cells were fixed with paraformaldehyde (4%) for 20 min at room temperature and washed three times with PBS for 5 min under stirring. Hoechst staining (Sigma Aldrich) was added to visualize cell nuclei. Samples were evaluated using a Nikon Eclipse 50i microscope (Nikon Instruments Inc, Melville, NY). In addition, an HPLC analysis was realized to determine the modulation of intracellular PTX concentration after PTX and Tripalm-NPs-PTX exposure (Supplementary section 4).

### Multidrug resistance inhibition

The ability to overcome MDR caused by P-gp using PTX-loading NPs was analyzed following Baek *et al*.^28^. Firstly, Tripalm-NPs-PTX and Tripalm-NPs-MAC-PTX were selected to determine cytotoxicity (see above) in HCT-15 (resistant) and T84 (sensitive) cells (5 × 10^3^ cells per well) in comparison to free PTX. Blank NPs were used as a control. In addition, cells were treated with the P-gp inhibitor Verapamil (Sigma Aldrich) (14.3 µM) 24 h before the administration of free PTX, Tripalm-NPs-PTX and Tripalm-NPs-MAC-PTX and replaced with fresh medium. In these combined treatments, cells treated only with verapamil were used as a control. In addition, Rhodamine retention was assayed by flow cytometry. HCT-15 cells were seeded in a 6-well plate (7 × 10^4^ cells per well) and after 24 h incubation, blank Tripalm-NPs, Tripalm-NPs-MAC and Verapamil were added at the same doses as the previous assay. After 24 h, Rhodamine 123 (Sigma Aldrich), a P-gp substrate, was added (1.3 µM) for 30 min, and then the medium was replaced by fresh medium with the P-gp inhibitor. Analyses were carried out immediately and after 30, 60 and 90 min, in order to assess Rhodamine release. Photographs were also taken with a fluorescent microscope at 30 minutes of Rhodamine release under the same conditions as the previous assay.

### Multicellular tumor spheroids analysis

Multicellular tumor spheroids (MTS) were generated as previously described^53^. Firstly, A549 and MCF7 cells (250 and 4 × 10^3^ cells per well, respectively) were seeded in a 96-well plate coated with 1% (w/v) agarose. In order to enhance cell aggregation, plates were centrifuged at 800 x g for 5 minutes, and then incubated at 37 °C in a 5% CO_2_ atmosphere for 4 days. Afterwards, MTS were exposed to PTX, blank Tripalm-NPs and Tripalm-NPs-PTX to reach the PTX IC_50_ and the equivalent of NPs, replacing 100 µl of medium by fresh medium with the corresponding treatment. This process was carried out twice every 48 h, and eventually, 100 µl of medium was replaced by fresh medium without treatment. Untreated MTS were used as a control. MTS were monitored by inverted phase-contrast microscopy, measuring the longest and shortest diameter (every 2 days). Median relative volume (V, mm3) was determined by the formula: V = a.b^2^. π/6 (where a is the longest diameter and b is the shortest diameter). In addition, to compare the apoptosis induced by Tripalm-NPs-PTX and free PTX in MTS, we used a TUNEL assay (TUNEL kit, Roche, Mannheim, Germany). MTS were treated as above, and at day 4 they were collected and fixed with 4% paraformaldehyde, for 3 h at room temperature. Then, the TUNEL assay was performed following the protocol of the manufacturer, and cell nuclei were counterstained with Hoechst. Fluorescence images were captured using confocal microscopy (Nikon A1, Nikon Corporation, Tokyo, Japan).

### Cancer stem cell assay

Breast and lung cancer stem cells (CSCs) were obtained from MCF7 and A549 respectively, following Hu *et al*.^54^ and real-Time PCR was carried out to asses CSCs phenotype (supplementary section 5). For the cytotoxicity assay, CSCs were collected on day 13, centrifuged at 1600 rpm for 10 min, and disaggregated with Tripsin/EDTA. Then, they were seeded in a 96-well plate (1 × 10^3^ cells/ well) and incubated in induction medium (100 μl) for 24 h. Ten μl of medium with the corresponding treatment (PTX and Tripalm-NPs-PTX at different concentrations) were added and cells were incubated for 72 h and then, 10 μl of cell counting kit-8 (CCK-8, Dojindo, Japan) were added for 3 h at 37 °C in a 5% CO_2_ atmosphere. OD was then measured with a Titertek multiscan colorimeter (Flow) at 450 nm. Cell viability (%) was calculated as described above.

### Statistical analysis

Student’s t-test and ANOVA was used (SPSS 7.5 software, Chicago, IL). Data are expressed as mean ± SD. Differences were considered statistically significant at a P-value ≤ 0.001.

## Electronic supplementary material

Supplementary Materials

## References

[1] Gornstein E, Schwarz TL (2014). The paradox of paclitaxel neurotoxicity: mechanisms and unanswered questions. Neuropharmacology.

[2] Marupudi NI (2007). Paclitaxel: a review of adverse toxicities and novel delivery strategies. Expert. Opin. Drug Saf..

[3] Surapaneni MS, Das SK, Das NG (2012). Designing paclitaxel drug delivery systems aimed at improved patient outcomes: current status and challenges. ISRN Pharmacol..

[4] Feng, L. & Mumper, R. J. A critical review of lipid-based nanoparticles for taxane delivery. *Cancer Lett.* **334**, 157–175 (2013). 10.1016/j.canlet.2012.07.006 PMC3485436 22796606

[5] Kundranda, M. N. & Niu, J. Albumin-bound paclitaxel in solid tumors: clinical development and future directions. *Des. Devel. Ther.* **9**, 3767–3777 (2015). 10.2147/DDDT.S88023 PMC4521678 26244011

[6] Cirri M, Bragagni M, Mennini N, Mura P (2012). Development of a new delivery system consisting in “drug–in cyclodextrin–in nanostructured lipid carriers” for ketoprofen topical delivery. Eur. J. Pharm. Biopharm..

[7] Wissing SA, Kayser O, Muller RH (2004). Solid lipid nanoparticles for parenteral drug delivery. Adv. Drug. Deliv. Rev..

[8] Geszke-Moritz M, Moritz M (2016). Solid lipid nanoparticles as attractive drug vehicles: composition, properties and therapeutic strategies. Mater. Sci. Eng. C.

[9] Hitzman CJ, Elmquist WF, Wiedmann TS (2006). Development of a respirable, sustained release microcarrier for 5-fluorouracil II: *In vitro* and *in vivo* optimization of lipid coated nanoparticles. J. Pharm. Sci..

[10] Reddy LH, Vivek K, Bakshi N, Murthy RS (2006). Tamoxifen citrate loaded solid lipid nanoparticles (SLN): preparation, characterization, *in vitro* drug release, and pharmacokinetic evaluation. Pharm. Dev. Technol..

[11] Ucisik MH (2015). S-layer fusion protein as a tool functionalizing emulsomes and CurcuEmulsomes for antibody binding and targeting. Colloids Surf. B Biointerfaces.

[12] Bondi ML (2015). Lipid nanocarriers containing sorafenib inhibit colonies formation in human hepatocarcinoma cells. Int. J. Pharm..

[13] Kuo YC, Chao IW (2016). Conjugation of melanotransferrin antibody on solid lipid nanoparticles for mediating brain cancer malignancy. Biotechnol. Prog..

[14] Cavalli R, Caputo O, Gasco MR (2000). Preparation and characterization of solid lipid nanospheres containing paclitaxel. Eur. J. Pharm. Sci..

[15] Serpe L (2004). Cytotoxicity of anticancer drugs incorporated in solid lipid nanoparticles on HT-29 colorectal cancer cell line. Eur. J. Pharm. Biopharm..

[16] Elkharraz K (2006). Paclitaxel-loaded microparticles and implants for the treatment of brain cancer: preparation and physicochemical characterization. Int. J. Pharm..

[17] Ernsting MJ, Murakami M, Roy A, Li SD (2013). Factors controlling the pharmacokinetics, biodistribution and intratumoral penetration of nanoparticles. J. Control. Release.

[18] Lee M-K, Lim S-J, Kim C-K (2007). Preparation, characterization and *in vitro* cytotoxicity of paclitaxel-loaded sterically stabilized solid lipid nanoparticles. Biomaterials.

[19] Zheng J, Wan Y, Elhissi A, Zhang Z, Sun X (2014). Targeted paclitaxel delivery to tumors using cleavable PEG-conjugated solid lipid nanoparticles. Pharm. Res..

[20] Yuan H (2008). Cellular uptake of solid lipid nanoparticles and cytotoxicity of encapsulated paclitaxel in A549 cancer cells. Int. J. Pharm..

[21] Kurkov SV, Loftsson T (2013). Cyclodextrins. Int. J. Pharm..

[22] Bilensoy E, Gurkaynak O, Dogan AL, Hincal AA (2008). Safety and efficacy of amphiphilic beta-cyclodextrin nanoparticles for paclitaxel delivery. Int. J. Pharm..

[23] Baek JS, Cho CW (2013). 2-Hydroxypropyl-beta-cyclodextrin-modified SLN of paclitaxel for overcoming p-glycoprotein function in multidrug-resistant breast cancer cells. J. Pharm. Pharmacol..

[24] Paul S (2013). Multiple biological properties of macelignan and its pharmacological implications. Arch. Pharm. Res..

[25] Kapse-Mistry, S., Govender, T., Srivastava, R. & Yergeri, M. Nanodrug delivery in reversing multidrug resistance in cancer cells. *Front. Pharmacol*. **5** (2014). 10.3389/fphar.2014.00159 PMC4090910 25071577

[26] Qiang F (2010). Effect of maceligan on the systemic exposure of paclitaxel: *in vitro* and *in vivo* evaluation. Eur. J. Pharm. Sci..

[27] Gupta P, Garg T, Tanmay M, Arora S (2015). Polymeric Drug-Delivery Systems: Role in P-gp Efflux System Inhibition. Crit. Rev. Ther. Drug Carrier Syst..

[28] Baek JS, Cho CW (2015). Controlled release and reversal of multidrug resistance by co-encapsulation of paclitaxel and verapamil in solid lipid nanoparticles. Int. J. Pharm..

[29] Benavente J (2010). Modification of a regenerated cellulose membrane with lipid nanoparticles and layers. Nanoparticle preparation, morphological and physicochemical characterization of nanoparticles and modified membranes. J. Memb. Sci..

[30] Salvia-Trujillo L, Rojas-Graü MA, Soliva-Fortuny R, Martín-Belloso O (2013). Effect of processing parameters on physicochemical characteristics of microfluidized lemongrass essential oil-alginate nanoemulsions. Food Hydrocoll..

[31] Marquele-Oliveira F (2016). Physicochemical characterization by AFM, FT-IR and DSC and biological assays of a promising antileishmania delivery system loaded with a natural Brazilian product. J. Pharm. Biomed. Anal..

[32] Marquele-Oliveira F (2010). Development of nitrosyl ruthenium complex-loaded lipid carriers for topical administration: improvement in skin stability and in nitric oxide release by visible light irradiation. J. Pharm. Biomed. Anal..

[33] Bernabeu E (2014). Paclitaxel-loaded PCL-TPGS nanoparticles: *in vitro* and *in vivo* performance compared with Abraxane(R). Colloids Surf. B Biointerfaces.

[34] Pizzol CD (2014). Influence of surfactant and lipid type on the physicochemical properties and biocompatibility of solid lipid nanoparticles. Int. J. Environ. Res. Public Health.

[35] Subik K (2010). The expression patterns of ER, PR, HER2, CK5/6, EGFR, Ki-67 and AR by immunohistochemical analysis in breast cancer cell cines. Breast Cancer.

[36] Acevedo-Morantes CY, Acevedo-Morantes MT, Suleiman-Rosado D, Ramirez-Vick JE (2013). Evaluation of the cytotoxic effect of camptothecin solid lipid nanoparticles on MCF7 cells. Drug Deliv..

[37] Martin-Banderas L (2015). *In vitro* and *in vivo* evaluation of Delta(9)-tetrahidrocannabinol/PLGA nanoparticles for cancer chemotherapy. Int. J. Pharm..

[38] Sahu PK, Mishra DK, Jain N, Rajoriya V, Jain AK (2015). Mannosylated solid lipid nanoparticles for lung-targeted delivery of Paclitaxel. Drug Dev. Ind. Pharm..

[39] Park K (2010). To PEGylate or not to PEGylate, that is not the question. J. Control. Release.

[40] Fan T (2014). Design and evaluation of solid lipid nanoparticles modified with peptide ligand for oral delivery of protein drugs. Eur. J. Pharm. Biopharm..

[41] Rivolta I (2011). Cellular uptake of coumarin-6 as a model drug loaded in solid lipid nanoparticles. J. Physiol. Pharmacol..

[42] Salomon JJ, Ehrhardt C (2011). Nanoparticles attenuate P-glycoprotein/MDR1 function in A549 human alveolar epithelial cells. Eur. J. Pharm. Biopharm..

[43] Koziara JM, Whisman TR, Tseng MT, Mumper RJ (2006). *In-vivo* efficacy of novel paclitaxel nanoparticles in paclitaxel-resistant human colorectal tumors. J. Control. Release.

[44] Kang A, Seo HI, Chung BG, Lee SH (2015). Concave microwell array-mediated three-dimensional tumor model for screening anticancer drug-loaded nanoparticles. Nanomedicine.

[45] Prados J (2008). Combined therapy using suicide gef gene and paclitaxel enhances growth inhibition of multicellular tumour spheroids of A-549 human lung cancer cells. Int. J. Oncol..

[46] Swaminathan SK (2013). CD133-targeted paclitaxel delivery inhibits local tumor recurrence in a mouse model of breast cancer. J. Control. Release.

[47] Muntimadugu E, Kumar R, Saladi S, Rafeeqi TA, Khan W (2016). CD44 targeted chemotherapy for co-eradication of breast cancer stem cells and cancer cells using polymeric nanoparticles of salinomycin and paclitaxel. Colloids Surf. B Biointerfaces.

[48] Sadeghi-aliabadi H, Asghari G, Mostafavi SA, Esmaeili A (2009). Solvent optimization on Taxol extraction from Taxus baccata L., using HPLC and LC-MS. DARU.

[49] Hou D, Xie C, Huang K, Zhu C (2003). The production and characteristics of solid lipid nanoparticles (SLNs). Biomaterials.

[50] Vazquez MI (2011). Functionalized lipid nanoparticles-cellophane hybrid films for molecular delivery: preparation, physicochemical characterization, and stability. J. Pharm. Sci..

[51] Perazzoli, G., *et al*. Temozolomide Resistance in Glioblastoma Cell Lines: Implication of MGMT, MMR, P-Glycoprotein and CD133 Expression. *PLoS One* **10**(2015). 10.1371/journal.pone.0140131 PMC4598115 26447477

[52] Evans, B. C., *et al*. *Ex vivo* red blood cell hemolysis assay for the evaluation of pH-responsive endosomolytic agents for cytosolic delivery of biomacromolecular drugs. JoVE e50166 (2013). 10.3791/50166 PMC3626231 23524982

[53] Ho WY, Yeap SK, Ho CL, Rahim RA, Alitheen NB (2012). Development of multicellular tumor spheroid (MCTS) culture from breast cancer cell and a high throughput screening method using the MTT assay. PLoS One.

[54] Hu K (2015). Hyaluronic acid functional amphipathic and redox-responsive polymer particles for the co-delivery of doxorubicin and cyclopamine to eradicate breast cancer cells and cancer stem cells. Nanoscale.

