# Supplementary material for: Tripalmitin nanoparticle formulations significantly enhance paclitaxel antitumor activity against breast and lung cancer cells in vitro
Source: Sci Rep. 2017 Oct 18;7:13506. doi: 10.1038/s41598-017-13816-z (PMC5647375; doi:10.1038/s41598-017-13816-z)
Supplement: Supplementary file 1 — Supplementary Materials [file 41598_2017_13816_MOESM1_ESM.pdf]

Tripalmitin nanoparticle formulations significantly enhance paclitaxel antitumor activity against breast and lung cancer cells in vitro

María Carmen Leiva[a,b,c], Raúl Ortiz[a,d], Rafael Contreras-Cáceres[e], Gloria Perazzoli[a], Iryna Mayevych[e], Juan Manuel López-Romero[e], Francisco Sarabia[e], Jose Manuel Baeyens[f], Consolación Melguizo[a,b,c,*], Jose Prados[a,b,c,*]

[a]Institute of Biopathology and Regenerative Medicine (IBIMER), Center of Biomedical Research (CIBM), University of Granada, 18100 Granada, Spain

[b]Department of Anatomy and Embryology, Faculty of Medicine, University of Granada. 18071, Granada, Spain

[c]Biosanitary Institute of Granada (ibs. GRANADA), SAS-Universidad de Granada, 18014 Granada, Spain

[d]Department of Health Science, University of Jaén, 23071 Jaén, Spain

[e]Department of Organic Chemistry, Faculty of Science. University of Málaga, 29071, Málaga, Spain;

[f]Department of Pharmacology, Institute of Neuroscience, Biomedical Research Center (CIBM), University of Granada, 18100, Granada, Spain

[*]These authors contributed equally to this work.

Correspondence: Juan Manuel López-Romero. Department of Organic Chemistry, Faculty of Science. University of Málaga, Málaga, Spain.

Email jmromero@uma.es

Supplementary Tables

Table S1. PTX-loaded Tripalmitin NPs chemical composition

Entry	**PTX (mg)**	**TP (g)**	**Saline (mL)**	**Aditive (mg)**		**Homog (min)**	**nB (g)**	**TW (g)**	**PC (g)**	**V_{tot} (mL)**
1	0.4	0.3	15	--		30	0.17	0.85	0.15	15
2	0.4	0.3	15	MAC	0.4	30	0.17	0.85	0.15	15
3	0.4	0.3	30	MAC	6.0	30	0.17	0.85	0.15	15
4	0.4	0.3	15	MAC	20.0	15	0.17	0.85	0.15	15
6	0.4	0.3	15	OEG	20.0	15	0.17	0.85	0.15	15
7	0.4	0.3	15	OEG	23.0	30	0.17	0.85	0.15	15
8	0.4	0.3	15	FITC	25.0	15	0.17	0.85	0.15	15
9	0.4	0.3	15	βCD	0.4	30	0.17	0.85	0.15	15
10	0.4	0.3	15	βCD	20.0	15	0.17	0.85	0.15	15
11	0.4	0.3	15	FITC	25.0	15	0.17	0.85	0.15	15

PTX, Paclitaxel; TP, Tripalmitin; Homog, homogenization; nB, *n*-butanol; TW, Tween® 80; PC, *L*–α-phosphatidylcholine; V_{tot}, total volume; MAC, macelignan; OEG, hexa(ethylene glycol); βCD, β-cyclodextrin.

Supplementary Figures

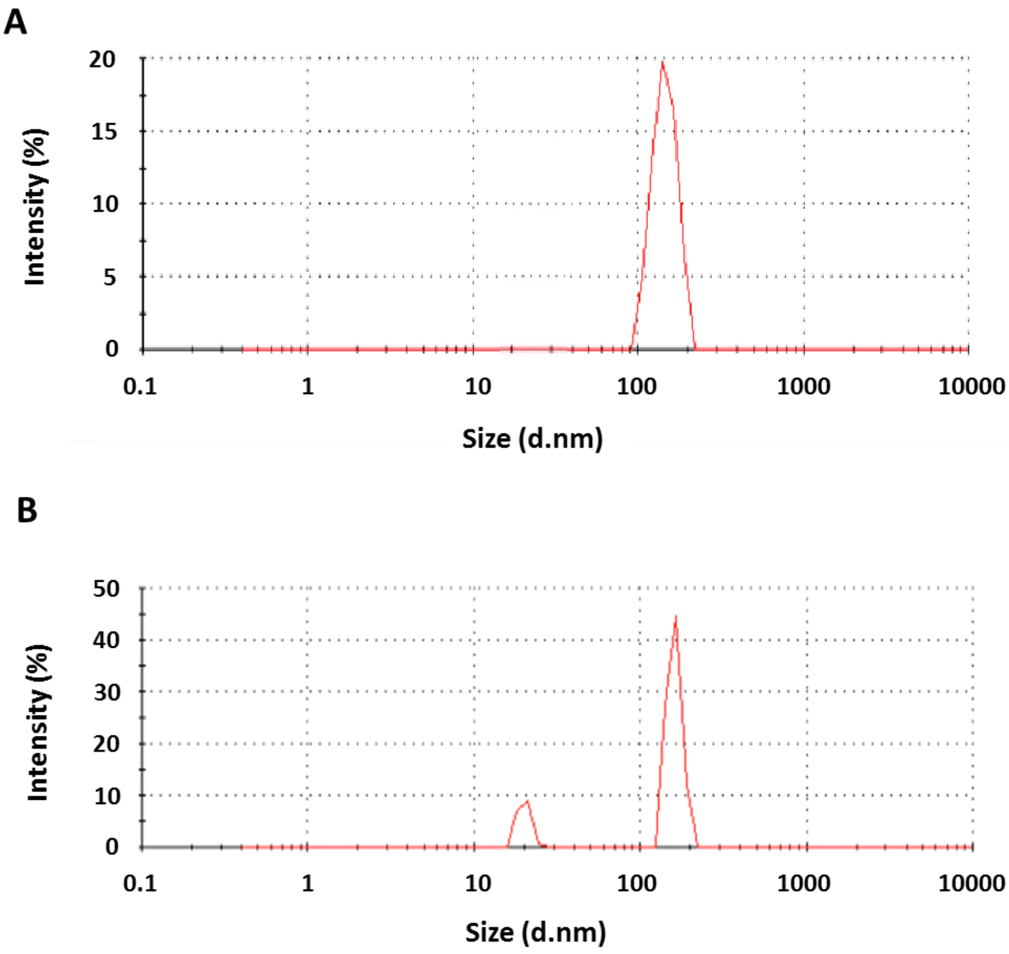

Figure S1. Zeta Potential analyses of of representative samples of Tripalm-NPs-PTX. (A) Distribution Result: Peak 1: 190.2 d.nm /100% (B) Distribution Result: Peak 1: Mean 29.8 d.nm / Area 18.1%; Peak 2: Mean 195.8 d.nm / Area 81.9%.

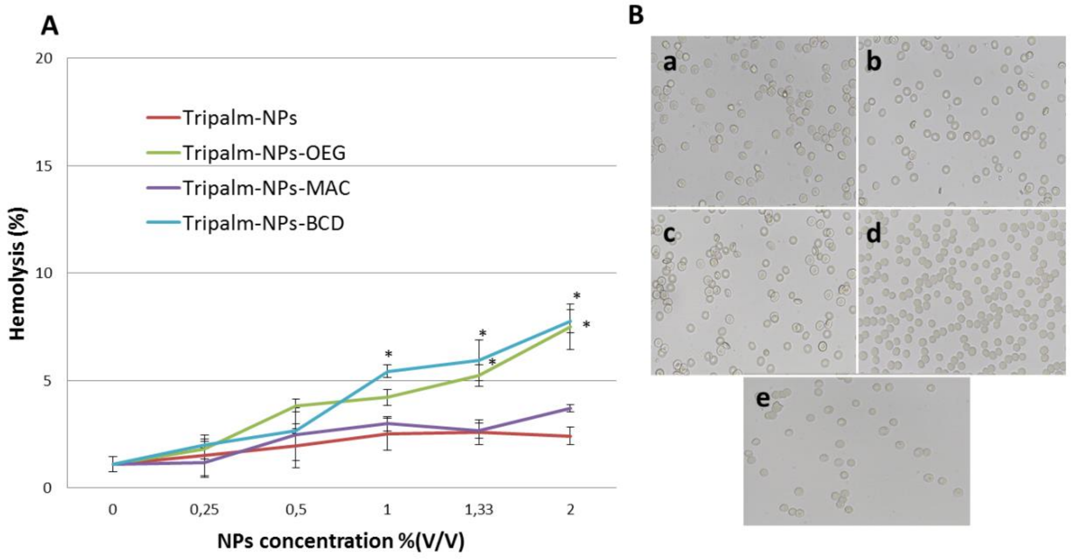

Figure S2. Tripalmitin NPs hemolysis. (A) Erythrocytes from human blood were exposed to Tripalm-NPs and modified Tripalm-NPs (Tripalm-NPs-MAC, Tripalm-NPs-OEG and Tripalm-NPs-βCD) at different concentrations during 2 hours. Hemoblobin was measured at a wavelength= 450 nm. Triton X-100 was used as control representing the 100% hemolysis. Data represent the mean value ± SD of quadruplicate experiences. (*) Significant differences ($p<0.001$) between modified Tripalm-NPs and Tripalm-NPs. (B) Morphology of the erythrocytes from human blood exposed to Tripalm-NPs and modified Triplam-NPs at concentration 2% (V/V) during 2 hours. (a) Phosphate buffer (pH 7.4) (negative control); (b) Tripalm-NPs; (c) Tripalm-NPs-MAC; (d) Tripalm-NPs-βCD and (e) Tripalm-NPs-OEG. Most of the erythrocytes presented a discoid shape after all the treatments. Erythrocytes with echinocyte conformation were observed (specially with Tripalm-NPs and Tripalm-NPs-βCD). Stomatocytes were also observed after incubation with Tripalm-NPs-MAC and Tripalm-NPs-OEG. Magnification= 40X.

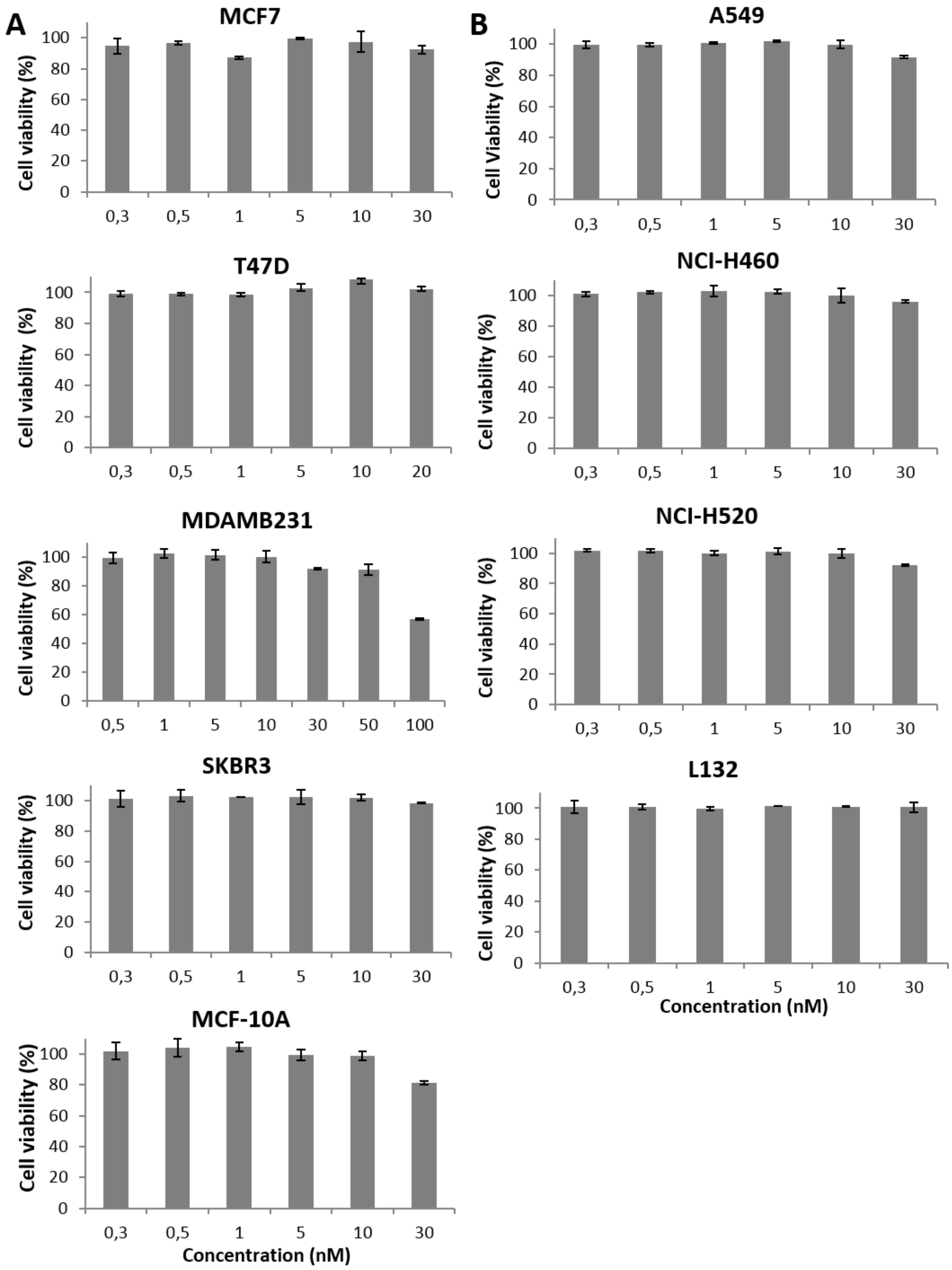

Figure S3. Cytotoxicity of Tripalm-NPs. Cell viability (%) of human breast tumor and normal cells (A) and human lung tumor and normal cells (B) was tested after treatment with Tripalm-NPs. Cells were exposed to a wide range of Tripalm-NPs concentrations equivalent to the ones used in the proliferation assay, over 96 hours. Results are expressed as cell viability (%). Data represent the mean value ± SD of quadruplicate cultures. Only a light toxicity could be observed in MCF-10A cells at the highest concentration of NPs and in MDAMB231 cells at the concentration of 100 µM (drug dose had to be very high to reach the IC_{50}).

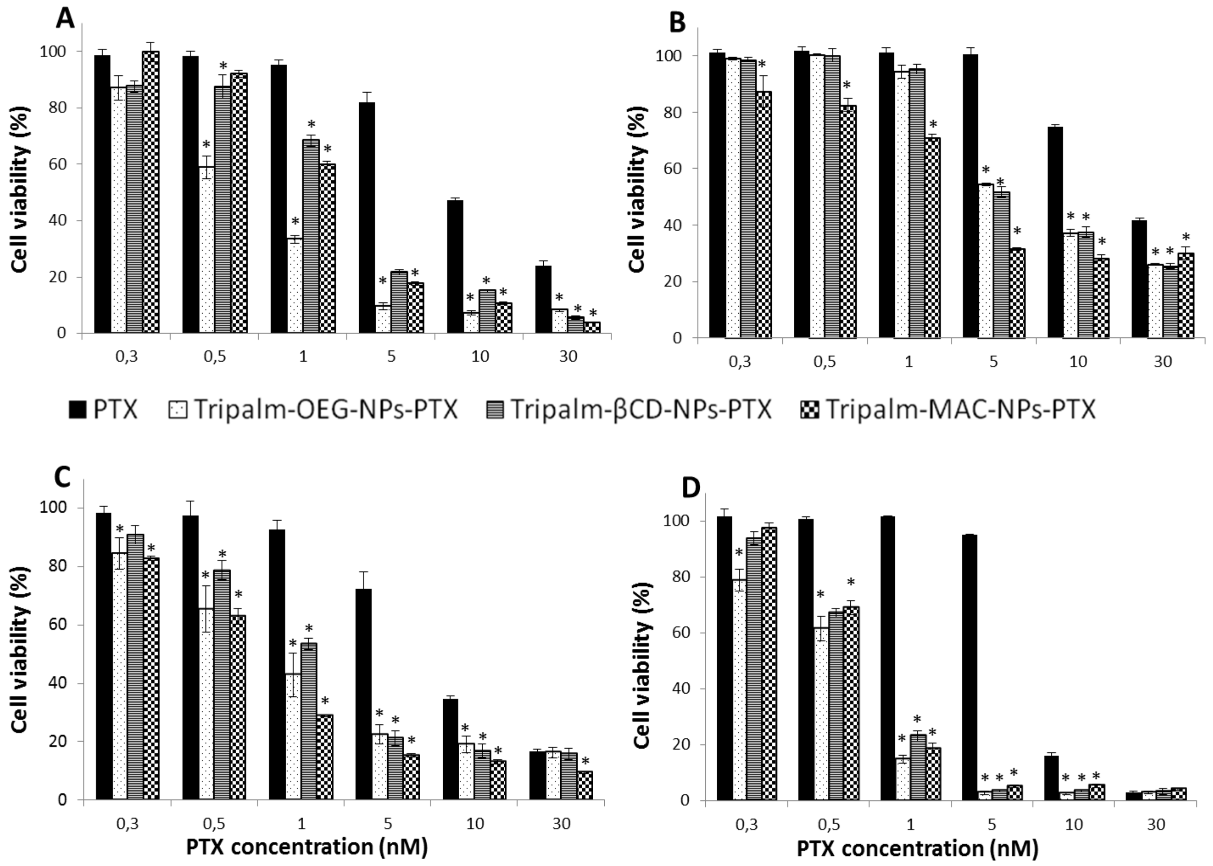

Figure S4. Cytotoxicity of modified-Tripalm-NPs-PTX with MAC, OEG and βCD. Cell viability (%) of MCF7 (A) and MCF-10A (B) (breast tumor and normal cells, respectively) and A549 (C) and L132 (D) (lung cancer and normal cells, respectively) after treatment with modified Tripalm-NPs-PTX and free PTX. Data represent the mean value ± SD of quadruplicate cultures. (*) Significant differences ($p \leq 0.001$) between free PTX and modified Tripalm-NPs-PTX.

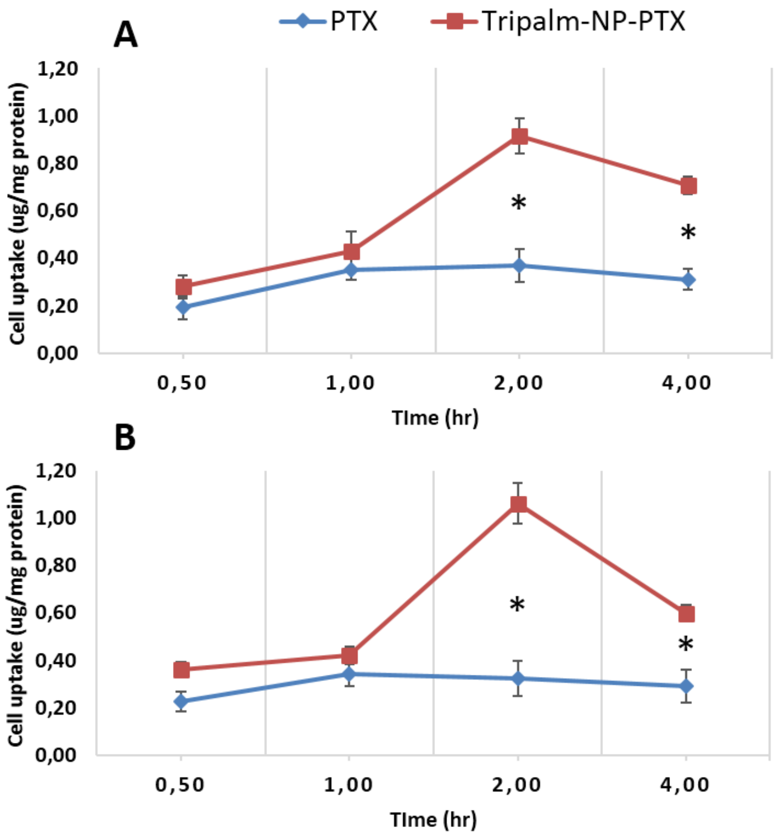

Figure S5. Uptake of PTX assessed by HPLC. A549 (A) and MCF7 (B) cells treated with PTX free (500 nM) and Tripalm-NP-PTX (equivalent concentration of PTX). Tripalm-NPs-PTX treatment induced a significant increase of PTX uptake value in A549 cells (0.91 ± 0.07 µg PTX/mg cell protein) in comparison to free PTX (0.37 ± 0.07 µg PTX/mg cell protein) after 2 hours. This difference was more evident in MCF7 cells (1.06 ± 0.08 µg versus 0.32 ± 0.07 µg of PTX/mg cell protein for Tripalm-NP-PTX and free PTX treatment, respectively). After 4 h, intracelular PTX was also higher in A-549 cells treated with Tripalm-NPs-PTX (0.71 ± 0.04 µg PTX /mg cell protein) than those exposed to free PTX (0.31 ± 0.04 µg PTX /mg cell protein). Similar results were observed in MCF-7 cells (0.60 ± 0.03 µg versus 0.29 ± 0.07 µg PTX/mg cell protein). These results indicated that the use of tripalmitin NPs increased the PTX incorporation inside both cancer cells. Data represent the mean value ± SD of triplicate cultures. (*) Significant differences ($p \leq 0.001$) between free PTX and Tripalm-NPs-PTX.

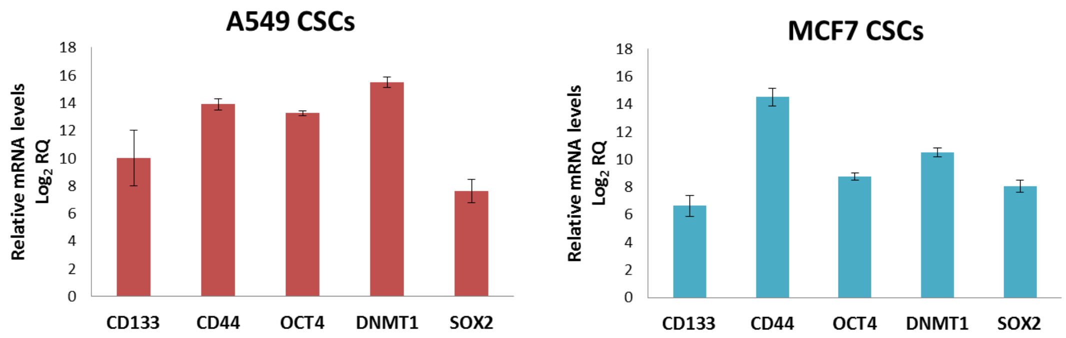

Figure S6. T-qPCR analysis of CSCs derived from MCF7 and A549 tumorspheres. Relative expression (RQ) of CD133, CD44, OCT4, DNMT1 and SOX2 mRNAs (CSCs phenotype) was validated through reverse transcription and RT-PCR. Data show log2 of the fold change relative to original A549 and MCF7 tumoral cells, representing mean ± SD of three samples.

Supplementary section

Section 1. Morphology analysis and size of PTX-loaded tripalmitin NPs

Nanoparticle morphology was characterized by SEM, AFM and Zeta Potential (ZP) measurements. For SEM analysis, spray-dried NPs were coated with gold for 3 min by sputtering (JEOL JCC-1100, JEOL Ltd., Madrid, Spain) before SEM recordings. The accelerating voltage ranged from 5 to 15 kV for scanning. SEM of the electrospun samples was performed on a JEOL JSM-840 JE (JEOL Ltd., Madrid, Spain) electron microscope. The surface topography was studied by Atomic Force Microscopy (AFM) (Veeco Instruments, Multimode AFM with Nanoscope V controller) equipped with a 12 μm EV-scanner. The Tapping ModeTM tip in air (42 N/m, 320 kHz) was used to scan the surface. WSxM 4.0 (Nanotec Electronic S.L.) software was used for the AFM image processing [28]. Samples of NPs emulsions were diluted with deionized water and spread on a mica sheet.

The Zeta Potential was used to monitor the diameter of the PTX-loaded Tripalm-NPs. Measurements were carried out using a Zetasizer Nano S (Malvern Instruments, Malvern UK) with a detection angle of 173°. The Nano S used a 4 mW He–Ne laser operating at a wavelength of 633 nm. The correlation function was used to calculate the Z-average (mean intensity). For Zeta Potential measurements, diluted samples (1 mg/mL) of NPs were prepared

by adding deionized water (100 mL to 1 mL of freshly prepared NPs emulsion). Then, samples were sonicated for 1 min in order to minimize the inter-particle interactions and remove air bubbles. Finally, tests were carried out at 25 ºC in duplicate.

Section 2. Chemical characterization of PTX-loaded tripalmitin NPs

Chemical characterization of PTX-loaded NPs was carried out by micro-Raman spectroscopy. The samples were prepared by depositing 0.1 mL of the dispersion onto a freshly cleaved glass plate and then drying at 20 ºC overnight in a desiccator ($CaCl_2$). For chemical composition studies of the NPs, isolated lipid PTX-loaded particles were chosen. The samples were analyzed at room temperature using a micro-Raman spectrophotometer (Renishaw Invia Reflex Raman, Madrid, Spain). The excitation source was an Ar^+ laser with an excitation wavelength of 514.5 nm. The spectra were recorded with a spatial resolution of 2μm and the 514.5nm exciting line of the Ar^+ laser of the Raman microscope.

Section 3. Hemocompatibilty assay

Samples of human blood (25 mL) from a healthy donor were collected in tubes with EDTA and centrifuged (500×g for 5 min) to obtain erythrocytes. After discarding the plasma, tubes were washed and filled with 150 mM NaCl (the same volume as the aspirate), mixed, and centrifuged again (500×g for 5 min). The supernatant was then aspirated and replaced with PBS at pH 7.4. Erythrocytes were diluted to 2% hematocrit (V/V) and then added (190 µL) to each well of a V-bottom 96-well plate. Blank NPs were added (at different concentrations and with 10 µl per well). The plate was incubated for 2 h at 37 °C under stirring (15 rpm) and then photographed at the highest concentration tested. Then the plate was centrifuged (500×g for 5 min) and supernatant (100 µl) was transferred to a new flat-bottomed 96-well plate. Triton X-100 (10 µL; 20 %) and phosphate buffer pH 7.4 (10 µl) were used as positive and negative controls respectively. The hemoglobin released from the erythrocytes was determined by a Titertek multiscan colorimeter (450 nm) (Flow, Irvine, California).

Section 4. Intracellular pharmacokinetics of PTX in A549 and MCF7 cell lines. A549 and MCF7 cells were seeded in a 6-well plate ($15x10^4$ cells/well) in 2 mL of complete DMEM. After 24-hour, the cells were treated with free PTX and Tripalm-NPs-PTX (500 nM) at 0.5, 1, 2 and 4

h. After remove the medium and following the protocol described in Li et al.[1], the cells were washed twice with PBS (2 ml) and resuspended in cell lysis buffer (800 µl/well) for 5 min. Then, the broken cells were collected and sonicated to completely disrupt cellular structures. The cell mixture was then mixed with of Methyl tert-butyl ether (500 µl) to extract PTX and docetaxel (15 µl) internal standard and shaked vigorously for 5 min. After centrifugation, the organic phase was collected and evaporated to dry. Samples were analyzed by HPLC as detailed Fernández-Peralbo et al.[2]

References

1. Li,M., Czyszczon, E.A. & Reineke, J.J. Delineating intracellular pharmacokinetics of paclitaxel delivered by PLGA nanoparticles. *Drug Deliv Transl Res*. **3**, 551-561 (2013).

2. Fernández-Peralbo, M.A., Priego-Capote, F., Luque de Castro, M.D., Casado-Adam, A., Arjona-Sánchez, A. & Muñoz-Casares, F.C. LC–MS/MS quantitative analysis of paclitaxel and its major metabolites in serum, plasma and tissue from women with ovarian cancer after intraperitoneal chemotherapy. *J Pharm Biomed Anal*. **91**,131-137 (2014).

Section 5. Cancer stem cell assay

Cells were cultured in low attachment 6-well plates with an induction medium consist in serum free medium composed of DMEM/ Nutrient Mixture F-12 Ham supplemented with EGF (20 ng/mL), bFGF (20 ng/mL), Heparin (4 µg/mL) (Sigma Aldrich), B27 (1X) (Gibco, Spain) and 1% of penicillin-streptomycin. Every 2-3 days a third part of the medium was replaced by fresh medium and tumorspheres being observed after 2-3 days. To carry out Real-Time PCR to asses CSCs phenotype, RNA (1 µg) (RNeasy Mini Kit, Qiagen, MD, USA) was reversed transcribed with M-MLV reverse transcriptase (Sigma, Italy). SYBR Green-based amplification (Applied Biosystems, Foster City, CA) was performed with the CFX96 Real-Time PCR Detection System (Bio-Rad, Italy), as previously we reported. Specific primer sequences were used to amplified SOX2, OCT4, DNMT1, CD133, CD44 and HPRT mRNA. The PCR cycling program was: 50°C (2min), 95°C (2 min), 45 cycles of denaturation at 95°C (30 s), annealing at 56°C (30

s), and extension at 72°C (40 s), followed by a melting curve analysis (range 56–95°C) with increments of 0.5°C/ 5 s to assess the primer specificity. The target transcripts were independently normalized to HPRT (housekeeping gene), and the RNA of the T0 cells was used as the calibration control. The results were expressed on a logarithmic scale as fold changes (FCs), with the 2–ΔΔCt method.

Real-Time PCR to assess CSCs phenotype: primer sequences.

**Gene**		**Primer sequence (5´→ 3´)**	**Bases**
SOX2	Forward	ATA ATA ACA ATC ATC GGC GG	20
	Reverse	AAA AAG AGA GAG GCA AAC TG	20
OCT4	Forward	GAT CAC CCT GGG ATA TAC AC	20
	Reverse	GCT TTG CAT ATC TCC TGA AG	20
DNMT1	Forward	CGT AAA GAA GAA TTA TCC GAG G	22
	Reverse	GTT TTC TAG ACG TCC ATT CAC	21
CD133	Forward	AAG CAT TGG CAT CTT CTA TG	20
	Reverse	TTT GCT CTG GAG TTT CAT TC	20
CD44	Forward	TTA TCA GGA GAC CAA GAC AC	20
	Reverse	ATC AGC CAT TGT GGA ATT TG	20
HPRT	Forward	TGA CAC TGG CAA AAC AAT GCA	21
	Reverse	GGT CCT TTT CAC CAG CAA GCT	21
